# How nanoscale protein interactions determine the mesoscale dynamic organisation of bacterial outer membrane proteins

**DOI:** 10.1038/s41467-018-05255-9

**Published:** 2018-07-20

**Authors:** Matthieu Chavent, Anna L. Duncan, Patrice Rassam, Oliver Birkholz, Jean Hélie, Tyler Reddy, Dmitry Beliaev, Ben Hambly, Jacob Piehler, Colin Kleanthous, Mark S. P. Sansom

**Affiliations:** 1 Department of Biochemistry, University of Oxford, South Parks Road, Oxford, OX1 5RJ UK; 2 Institut de Pharmacologie et Biologie Structurale, IPBS, Université de Toulouse, CNRS, UPS, 205 Route de Narbonne, Toulouse, 31400 France; 3 Department of Biology, University of Osnabrück, Barbarastraße 11, 49076 Osnabrück, Germany; 4 Mathematical Institute, University of Oxford, Andrew Wiles Building, Radcliffe Observatory Quarter (550), Woodstock Road, Oxford, OX2 6GG UK; 5 Present Address: Laboratoire de Biophotonique et Pharmacologie, UMR 7213 CNRS, Faculté de Pharmacie, Université de Strasbourg, 74 Route du Rhin, 67401 Illkirch, France; 6 Present Address: SEMMLE, Blue Boar Court, 9 Alfred St, Oxford, OX1 4EH UK; 7 Present Address: Theoretical Biology and Biophysics, T-6, Los Alamos National Laboratory, Los Alamos, NM 87525 USA

## Abstract

The spatiotemporal organisation of membranes is often characterised by the formation of large protein clusters. In *Escherichia coli*, outer membrane protein (OMP) clustering leads to OMP islands, the formation of which underpins OMP turnover and drives organisation across the cell envelope. Modelling how OMP islands form in order to understand their origin and outer membrane behaviour has been confounded by the inherent difficulties of simulating large numbers of OMPs over meaningful timescales. Here, we overcome these problems by training a mesoscale model incorporating thousands of OMPs on coarse-grained molecular dynamics simulations. We achieve simulations over timescales that allow direct comparison to experimental data of OMP behaviour. We show that specific interaction surfaces between OMPs are key to the formation of OMP clusters, that OMP clusters present a mesh of moving barriers that confine newly inserted proteins within islands, and that mesoscale simulations recapitulate the restricted diffusion characteristics of OMPs.

## Abstract (web summary)

In *Escherichia coli*, outer membrane protein (OMP) cluster and form islands, but the origin and behaviour of those clusters remains poorly understood. Here authors use coarse grained molecular dynamics simulation and show that their mesoscale simulations recapitulate the restricted diffusion characteristics of OMPs.

## Introduction

The dynamic organisation of membrane proteins is central to the biology of all cells. Membranes are crowded environments: it is estimated that ca. 25% of the cross-sectional area of a red blood cell membrane is occupied by proteins^1^, and this may be as high as ca. 40% in a bacterial outer membrane (OM)^2^. Such molecular crowding can result in dynamic clustering of membrane proteins^3–6^. Membrane protein clustering is observed both in eukaryotic^7^ and bacterial^8^ cell membranes, and is thought to be crucial to their organisation^9,10^ and biological function. In eukaryotic cells, interactions with the cytoskeleton underlying the cell membrane may also modulate the dynamic organisation of membrane proteins and lipids^11^, however, no such mechanism is apparent in bacterial cell membranes. We have previously demonstrated that clustering of outer membrane proteins (OMPs) in *Escherichia coli* results in the creation of asymmetric structures termed OMP islands^8^. These OMP islands are the size of eukaryotic organelles (diameter up to 0.5 µm) and contain hundreds (possibly thousands) of OMPs. The binary partitioning of OMP islands during cell division provides a means whereby OMPs are turned over in the OM, thus allowing Gram-negative bacteria to alter their OMP composition in response to a changing environment. OMP clustering also drives the clustering of inner membrane proteins when the two membranes become connected by an energised protein bridge^12^. The OM is an attractive target for novel antibiotics^13^, but to exploit this target greater knowledge of OMP behaviour and organisation is needed^14,15^. Here, we combine single molecule experimental approaches with computational simulation to show how the gap between nano- and meso-scale measurements on OMPs can be narrowed, in the process revealing principles about the dynamic organisation of bacterial OMPs.

A number of experimental methodologies allow us to probe the dynamics and organisation of crowded cell membranes^16^, e.g. the use of fluorescence correlation spectroscopy (FCS)^17^ or single particle tracking (SPT)^18^ to estimate protein diffusion rates in membranes. At the same time, e.g. high-speed AFM enables imaging of the dynamic organisation of OMPs in bacterial membranes in vitro^2^, and stimulated emission depletion (STED) can reveal the nanoscale dynamics of lipids in the membranes of living cells^19^. Taken together, these approaches provide descriptions of emergent complexities of the dynamic organisation of membranes at meso and micro scales. However, it remains challenging to link mesoscale organisation to atomic scale structural descriptions of the interactions between membrane proteins and lipids. In particular, we wish to know how OMP islands emerge as a consequence of atomic resolution interactions between membrane proteins, mediated by lipids.

Molecular simulations allow detailed exploration both of lipid/protein interactions of individual membrane proteins^20^ and the dynamic consequences of such interactions in terms of co-diffusion of lipids and proteins in membranes^21^. It is now possible to undertake such simulations of membranes on length and timescales, which begin to approach those observed experimentally^22^ whilst preserving aspects of the crowding and compositional complexity of cellular membranes^23^. This provides an opportunity to use simulations to more fully understand the molecular basis of mesoscale membrane organisation.

In this study, we employ large-scale simulations of OMP-containing membrane systems, at two levels of description, to characterise the process and consequences of membrane protein clustering. We thus develop a dynamic model of mesoscale organisation, which is derived from an underlying structural and dynamic description of membrane protein interactions as provided by the molecular simulations. This model permits exploration of the mesoscale both spatially (on a near-micrometre scale) and temporally (on a multi-millisecond scale). The simulations are used to emulate fluorescence data, enabling direct comparison with experimental data. By successfully bridging the gap between molecular level simulations and experiments, we thus obtain a mechanistic molecular interpretation of single molecule tracking data, revealing how dynamic clustering of OMPs results in the formation of mesoscale OM islands, which modulate the diffusional mobility of OMPs.

## Results

### OMPs form clusters at the nanoscale

Large-scale simulations are needed both to fully capture the dynamic behaviour of membrane proteins^24,25^ and to enable direct comparison with both in vitro and in vivo experiments. In the present work, we simulate the behaviour of OMPs in simple PE:PG bilayers devoid of the main lipid present in the outer leaflet of the OM, lipopolysaccharide (LPS). We contend for the following reasons that these simulations and associated in vitro experiments nevertheless provide fundamental insight into the behaviour of OMPs in the outer membrane of a Gram-negative bacterium. Past studies estimating the levels of LPS and OMPs in the outer membrane of *E. coli* suggest similar numbers of molecules (~10^6^). Total LPS has been estimated by radio-labelling methods^26,27^ while total OMP composition has been estimated by proteomics^28^. These previous studies therefore suggest that there are insufficient LPS molecules to encircle every OMP (although high affinity LPS binding has certainly been documented for a number of OMPs such as FhuA^29^). This probably explains why OMPs cluster in the OM of bacteria to produce OMP-rich regions^8,30,31^. Furthermore, OMPs at densities mimicking those found in the OM of *E. coli*, and incorporated into polymer supported membranes (PSM) composed solely of PE:PG, display diffusion coefficients and levels of restriction almost identical to those observed for OMPs in *E. coli*^8^. This observation is consistent with the hypothesis that the diffusion behaviour of OMPs in live bacteria is more influenced by promiscuous self-associations between OMPs rather than the presence of LPS. Moreover, the preponderance to β-barrel membrane protein self-association is not restricted to LPS-containing membranes; for example, the mitochondrial OMP VDAC is also seen to form densely packed clusters^32^. We therefore set out to simulate this behaviour of OMPs (in vitro) to better understand the inherent self-organisation of OMPs and how this impacts on their diffusion.

**Fig. 1:**
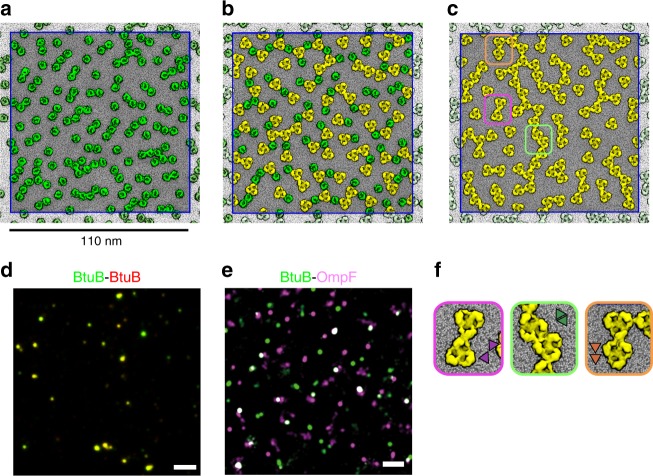
Simulations and experimental observations of OMPs compared. Final snapshots of 20 μs molecular simulations of **a** 144 molecules of BtuB (green); **b** 72 molecules of BtuB + 72 molecules of OmpF (yellow); and **c** 100 molecules of OmpF in a lipid bilayer (PE:PG 3:1) membrane and simulated at 323 K. For the OmpF system, the zoomed images (**f**) illustrate the three main configurations of OmpF association observed in the simulations: tip-to-tip (purple box), base-to-base (green box) and base-to-top (orange box) interactions of adjacent OmpF trimers (see main text and ref. ^2^). Further simulations demonstrating similar behaviour are shown in Supplementary Fig. 2. Clustering/co-localisation of **d** BtuB molecules (green and red; overlap in yellow) and of **e** BtuB (green) and OmpF (purple; overlap in white) molecules in supported bilayers (scale bar = 1 µm). It should be noted that **d**, **e** show under-labelled samples (we estimate less than 10% of BtuB molecules are linked to a fluorescent ColE9) in order to enable us to meaningfully distinguish single molecule trajectories

We have used a coarse-grained (CG) representation^33,34^ to model OMP-containing membranes on a ~100 nm length scale (Table 1), which approaches that studied experimentally^22^. The simulated systems thus contain 100 or more copies of an integral membrane protein, which enables us to sample formation of large-scale clusters. The lipid bilayer in these simulations is composed of a mixture of phosphatidylglycerol (PG) and phosphatidylethanolamine (PE), which matches that employed in in vitro experiments of OMP islands that largely recapitulates the behaviour of OMPs in vivo^8^. Addition of LPS in vitro did not significantly alter the clustering of OMPs nor their diffusive behaviour (Supplementary Figure 1). The protein density in the in vitro experiments was ca. 25% of the cross-sectional area of the membrane. In the simulations, the protein density was between 20 and 30%, so the simulations and in vitro experiment were comparable in terms of protein concentration. Moreover, the concentration of proteins in the model membranes corresponds to ca. 25%^8^ of the cross-sectional area of the membrane, which matches experimental estimates for both eukaryotic^1^ and bacterial cell membranes^2,26^. At the end of the 20 µs simulations, extended protein clusters had formed (Fig. 1 and Supplementary Figure 2). We simulated systems containing the vitamin B12 transporter BtuB or the porin OmpF alone, or an equimolar mixture of the two proteins. This matches the in vitro experimental studies which have focused upon these structurally and functionally well characterised OMPs. In all cases, cluster formation was observed (Fig. 1a–c) mimicking the co-localisation of these proteins observed in vitro in supported bilayers (Fig. 1d, e)^8^. These simulations were at a surface density of 10,000 OMPs µm^−2^ (See Table 1), comparable to that observed in bacterial OMs (e.g. see refs. ^2,35^). In studying BtuB and OmpF, we are simulating two OMPs, which represent the two extremes of OMP structures: monomeric and trimeric OMPs, respectively.

**Table 1:** Summary of CG–MD and mesoscale simulations performed

CG–MD simulations						
Simulation	Proteins	Lipids	Temperature (K)	Duration (µs)	Box dimensions at 20 µs (nm^3^)	Total number of particles
BtuB313	144 BtuB	28,888	313	20	109 × 109 × 12	1,331,636
BtuB323	144 BtuB	28,888	323	20	109 × 109 × 12	1,331,636
OmpF313	100 OmpF	26,832	313	20	112 × 112 × 12	1,370,636
OmpF323	100 OmpF	26,832	323	20	110 × 110 × 12	1,370,636
Mixed313	72 BtuB + 72 OmpF	25,448	313	20	112 × 112 × 12	1,377,536
Mixed323	72 BtuB + 72 OmpF	25,448	323	20	112 × 112 × 12	1,377,536
vBig	1152 BtuB + 1152 OmpF	407,168	323	2.5	446 × 446 × 12	22,040,576

Mesoscale simulations				
Simulation	Proteins	Duration (ms)	Box dimensions (µm^2^)	CG–MD simulation used for parameterisation
BtuB_interfaces	4900 BtuB	24	1 × 1	BtuB313 BtuB313
No_specific_interfaces	4900 BtuB^a^	24	1 × 1	BtuB313^a^
BtuB_1ms	2500 BtuB	1	0.5 × 0.5	BtuB313
Insert_25	2525 BtuB	1	0.5 × 0.5	BtuB313

^a^In this simulation, no specific interfaces were present, i.e. a pair of proteins upon encounter would interact regardless of their relative orientation

In the case of OmpF, protein–protein interactions underlying formation of clusters may be compared with direct observations using high-speed AFM^2^. As in AFM data, we observe the formation of three different classes of interaction between neighbouring OmpF trimers within larger clusters, namely: tip-to-tip, base-to-base and tip-to-base configurations (Fig. 1f and Supplementary Figure 2). Comparable OmpF–OmpF interactions have been reported in other CG simulations^2,36^, suggesting that this result is robust to minor changes in simulation protocol and reflects the underlying protein–protein interactions in a lipid bilayer environment. Visually comparing the three simulation systems (BtuB, BtuB + OmpF, and OmpF) it appears that the clusters are more linear in BtuB alone than in the presence of OmpF (Fig. 1a–c). This reflects the spatial distribution of protein interaction interfaces (see ref. ^8^ and Supplementary Figure 8). This clustering behaviour is distinct from that in other CG–molecular dynamics (MD) simulations of membrane proteins, e.g. of GPCRs^37^ and potassium channels^38^, which form clusters that are smaller or less linear, respectively.

**Fig. 2:**
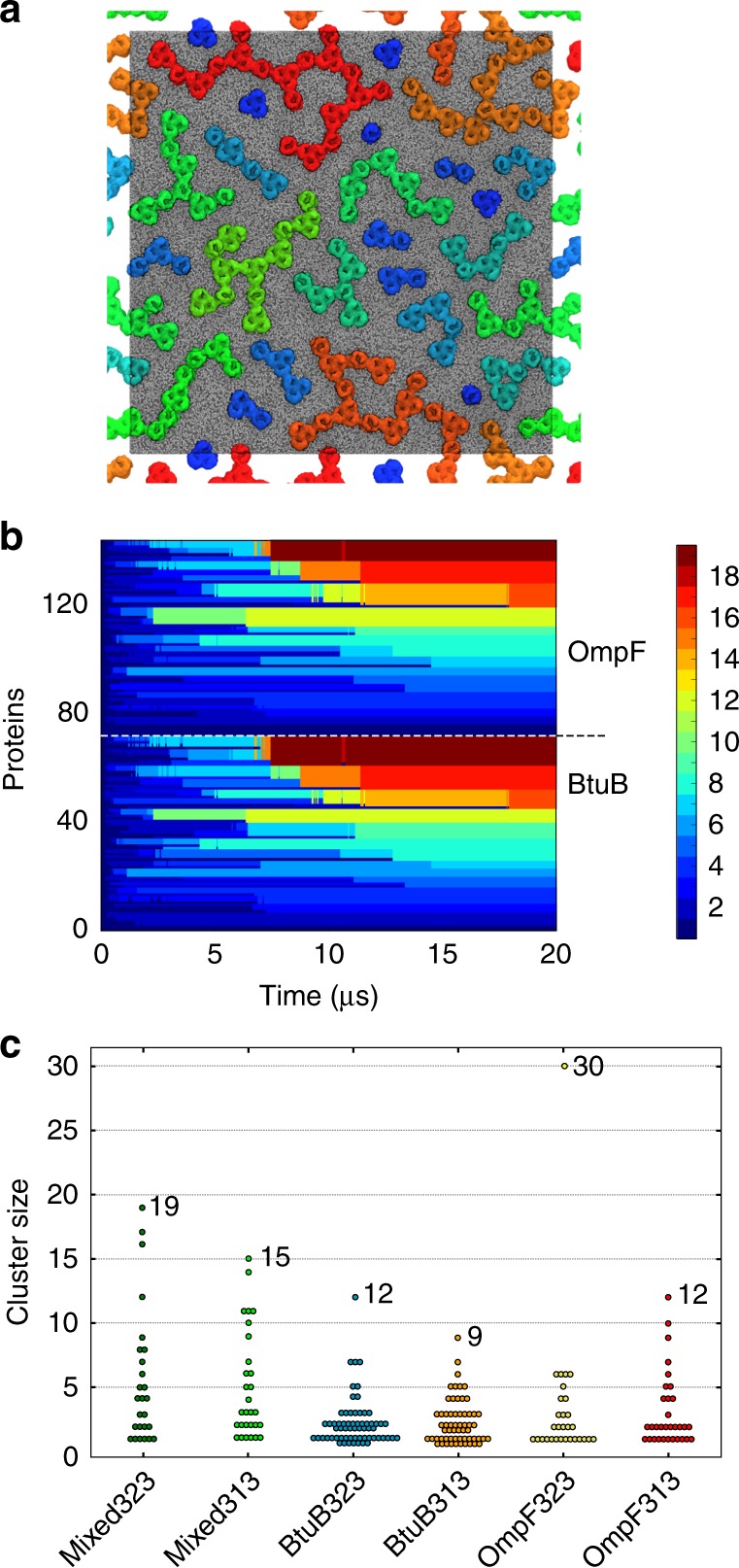
Clustering of OMPs in simulations. **a** Nanoscale clustering of BtuB and OmpF (Mixed323 simulation—see Table 1) at 20 µs. Proteins are coloured according to cluster membership on a blue (one of two molecules/cluster) to red (>16 molecules/cluster) scale. The colour scale, representing cluster size, is shown on the right side of the panel. **b** Evolution of cluster sizes over the course of the 20 µs Mixed323 simulation. Each horizonal line represents an individual protein, which is coloured according to cluster size on the same scale as in **a**. **c** Distribution of cluster sizes (at 20 µs) for all six simulations (see Table 1 for details, and Supplementary Figure 3 for snapshots and time evolution for all systems)

At the end of the simulations, a third of the protein molecules form small clusters (four or less) and the remainder are found in larger clusters ranging up to 30 protein molecules in the case of OmpF at 323 K (Fig. 2a, b, c). During the first 5 µs, OMP molecules interact to form dimeric and larger clusters (up to 4–5 proteins). Subsequently, these clusters coalesce to form higher order clusters (Fig. 2b and Supplementary Fig. 3). After the first 10 µs of simulation, the large clusters evolve more slowly (see Supplementary Movie 1 for an example of clustering for the BtuB + OmpF system). Thus, our simulations suggest that OMP islands^8^ are likely to provide a somewhat heterogeneous environment composed of a mixture of single OMPs and small clusters located within larger clusters which are anticipated to restrict their overall diffusional mobility. These interactions are mainly mediated by aromatic and hydrophobic residues on the surface of the proteins (see Supplementary Figure 4)^8^.

### Direct comparison of dynamics in simulations and experiments

**Fig. 3:**
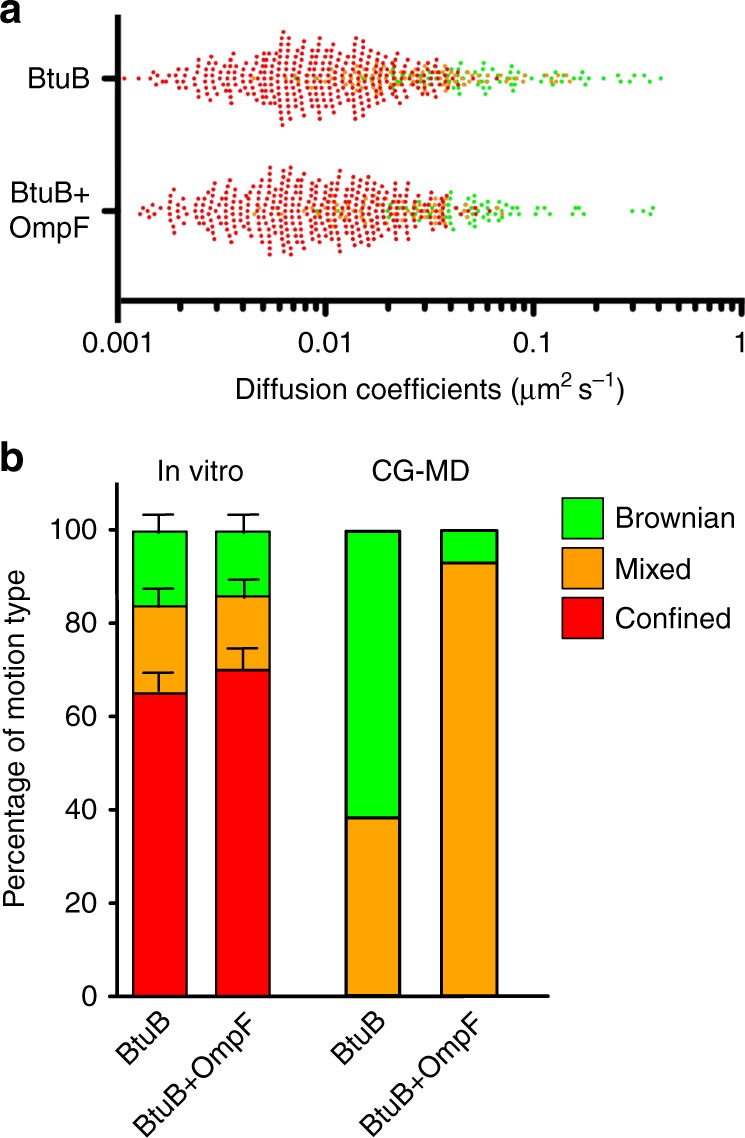
Comparing OMP diffusion in vitro and in simulations. **a** In vitro diffusion coefficients and classification of motion type (Brownian, green; confined, red; mixed, orange) for a supported bilayer containing a high area density (ca. 30%) of BtuB or BtuB and OmpF. See ref. ^8^ for further details. **b** Comparisons of the fraction of different classes of diffusional motion for in vitro systems and CG–MD simulations. The experimental single particles (error bars show standard error of mean, *n* = 3) and simulated OMP trajectories were both analysed using PaTrack^39^. The last 5 µs of the 20 µs simulations were used. Further details of the evolution of the diffusional motion as a function of time are available in Supplementary Figures 5, 6

The heterogeneity of cluster sizes in the simulated OMP-containing membranes would be expected to lead to a corresponding heterogeneity of diffusional dynamics, with higher diffusion rates for isolated protein molecules and those located in smaller clusters^3,8^. This may be compared with the diffusion coefficients obtained from in vitro experiments, which span almost three orders of magnitude (Fig. 3a). Although the timescales of experimental SPT (~100 ms) and these simulations (~10 µs) differ by four orders of magnitude, this provides an opportunity for comparison of the models and data. Analysis of in vitro single molecule tracking data (from TIRFM of supported OMP-containing bilayers; see ref. ^8^ for details) allows the diffusional behaviour of individual OMP molecules to be classified as Brownian, confined, or mixed (Fig. 3a, b). These diffusion modes fitted well with the different mobility behaviours that proteins displayed in the outer membrane in vivo^8^.

We therefore analysed the simulated OMP trajectories using the same analysis software (PaTrack^39^) as for the experimental data. This enables us to classify the modes of diffusion seen in the simulations in the same manner as for the experimental single tracking data, using a back-propagation neural network algorithm, trained and tested on synthetic and experimental data. Analysis of the experimental data demonstrated that when OMPs (either BtuB or BtuB + OmpF) are present at high densities in the in vitro membranes the dynamic behaviour (of BtuB) is predominantly ‘confined’ or ‘mixed’ rather than Brownian (see Fig. 3). This confinement is also visible for low densities of BtuB, and increases with time (see Supplementary Figure 5). In contrast, in the CG simulations diffusion is mainly Brownian, with ~40% ‘mixed’ in the BtuB alone simulations. This value is relatively constant over the course of the simulation (see Supplementary Figure 6). Inclusion of OmpF increases the proportion of mixed trajectories to ~90% (Fig. 3b and Supplementary Figure 6). This suggests a substantive slowing down of the BtuB proteins via their interactions with the larger and multivalent OmpF molecules. Nevertheless, the higher fraction of Brownian and mixed diffusion than observed in vitro indicates that the regimes captured at the nanoscale (via CG–MD) and at the mesoscale (via experiment) are not equivalent. This can also be seen if we compare the ranges of observed BtuB diffusion coefficients observed experimentally and in the CG simulations (see Supplementary Table 2 and Supplementary Figure 5B) where e.g. for the molecules undergoing Brownian diffusion, the experimental diffusion coefficients are ca. 0.1× those seen in the CG simulations. Furthermore, no confined movements are visible in the CG simulations, unlike the situation for the in vitro systems. This in turn suggests that the simulations may capture the early stages of island formation but not the confined behaviour seen in the single molecule tracking experiments.

**Fig. 4:**
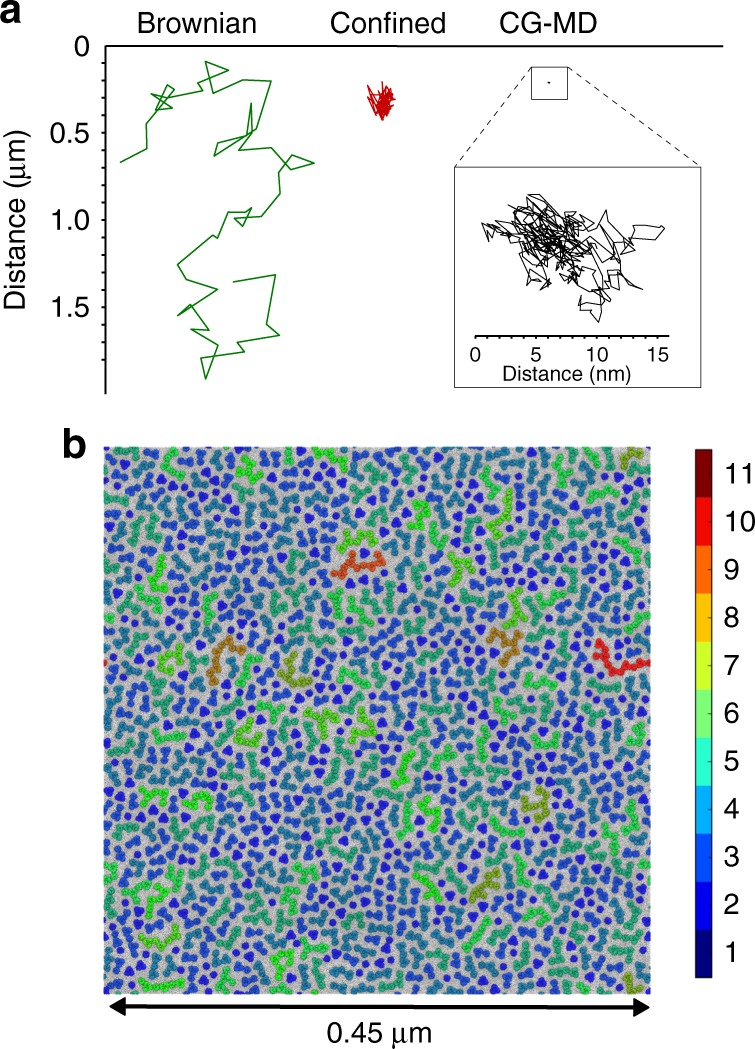
Increasing the spatial extent of CG–MD simulations. **a** Comparison of the spatial extent of two selected in vitro BtuB trajectories (Brownian, green; confined, red) and of a trajectory (black) selected from the BtuB323 CG–MD simulation. Note that the in vitro trajectories are collected over a timescale of seconds, whereas the CG–MD trajectory encompasses 20 µs. **b** Snapshot (2.5 µs) from a very large simulation of 1152 BtuB + 1152 OmpF on a length scale (0.5 µm) corresponding to that probed experimentally. The evolution of clustering behaviour is shown in Supplementary Figure 7. This simulation used 350,000 cpu.h per microsecond and generated 1.5 TB of data. The colour scale shows protein cluster size

This difference between simulation and experiment likely reflects difference in the size and the duration of simulations vs. the corresponding scales of the tracking experiments. As an example, we can display selected trajectories from tracking experiments and from a 1.3 M particle, 100 nm, 20 µs scale CG–MD simulation (Fig. 4a): it is clear that the full CG protein trajectory corresponds to, at best, a single point (i.e. pixel) from the in vitro tracking.

The clear implication from this comparison is that ‘longer and larger’ simulations are needed if we are to infer experimental diffusional behaviour (i.e. Brownian vs. confined) from molecular simulations. From our results, CG systems and in vitro data cannot be directly compared due to the large differences in spatial and temporal scale. Thus, trying to quantitatively compare the diffusion values extracted from CG–MD simulations with experimental diffusion coefficients remains challenging. It is in principle possible to simply run very large CG–MD simulations. For example, we simulated a 0.5 µm membrane patch (the vBig system containing 1152 BtuBs and 1152 OmpFs in Table 1 and Fig. 4b), thus directly mimicking the experiments. After 2.5 µs of simulation we saw equivalent cluster configurations to those observed in the smaller (114 protein) systems, with formation of clusters of up to 11 proteins (see Fig. 4 and Supplementary Figure 7). The very large size simulation reveals some nucleation points from which the clusters rapidly extend while in other regions of the membrane we only observe small clusters. This reinforces the heterogeneity of the system in the earlier stages of membrane protein clustering. However, such CG–MD simulations are very computationally demanding (using about a third of a million cpu.h per microsecond of simulation) and cannot reach the timescales we believe are necessary to observe full development of clustering and the ‘confined’ dynamic behaviour.

From this we may conclude that although large-scale CG–MD simulations enable us to initiate comparison between simulations and experimental studies of in vitro models of crowded membranes, we need to develop a higher-level mesoscale model if we are to link molecular interactions to the formation of OMP islands and the behaviour of OMPs in vivo.

### Development of a mesoscale model

**Fig. 5:**
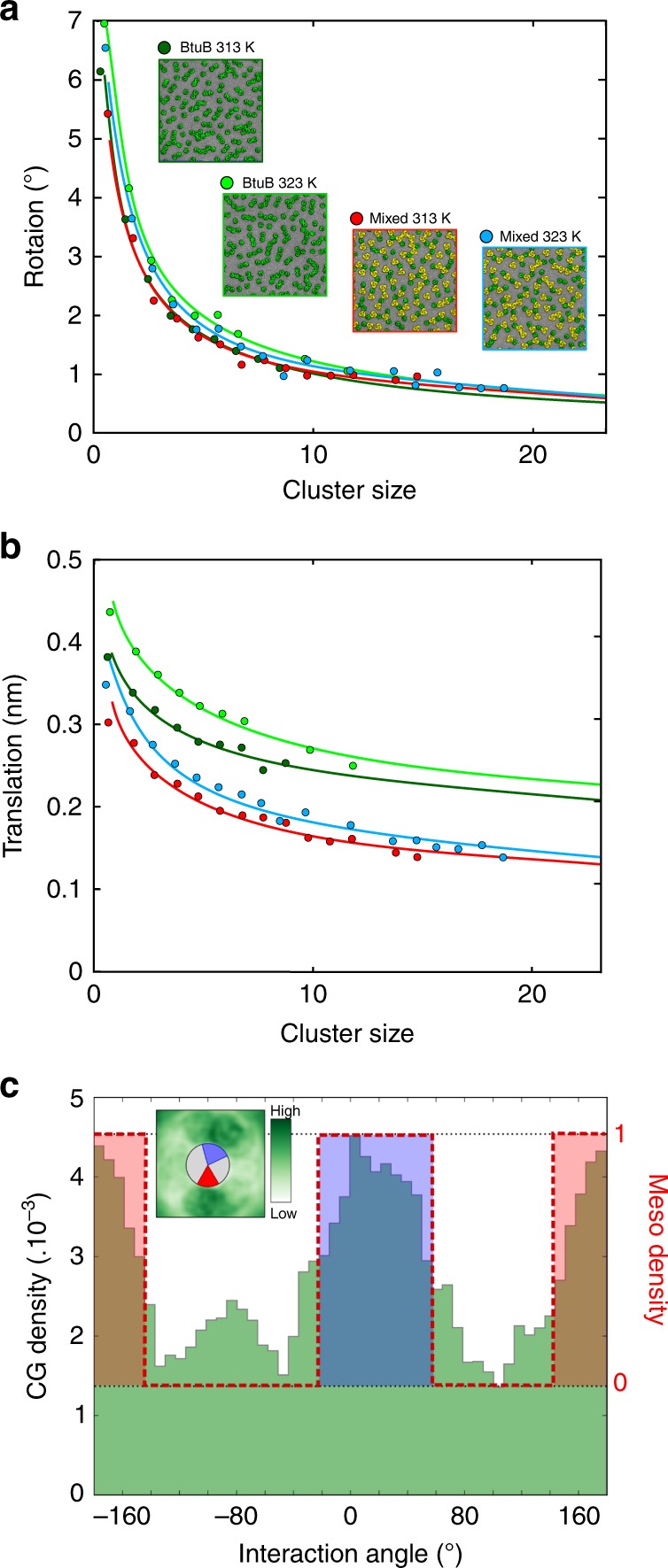
Analysis of CG–MD simulations and parameterisation of the mesoscale model. Standard deviation of the distributions of BtuB molecular rotations (**a**) and translation (**b**) as functions of cluster size for the four BtuB-containing simulations. Rotations and translations were collected for the duration of each simulation over the time interval of 10 ns, and were found to be distributed normally, with mean zero. The lines correspond to fitted power law functions used to parameterise the corresponding mesoscale models (see Supplementary Table 1 for details). **c** Frequency of occurrence (expressed as a density; see inset) of a neighbouring interacting BtuB molecule as a function of angle around a central BtuB molecule (from simulation BtuB313; also see Supplementary Figure 8). The two main peaks in the distribution, from −10° to 55° (blue) and from 155° to −155° (red), were used to define sticky patches of interaction between adjacent BtuB molecules in a mesoscale model (see main text for details)

We have developed a mesoscale model to describe the dynamics and interactions of OMPs in crowded membranes on more extended length and timescales than are feasible for CG–MD. As a proof of concept, we focused on the system containing only BtuB proteins as this system is well characterised experimentally. An important aspect of this model is that its parameters are derived from the dynamic behaviour observed in our large CG–MD simulations. Given the relatively small scale fluctuations of the OMP-containing bilayers, leading to deviations from planarity for this system (estimated to correspond to an RMSD height fluctuation of 4 nm on a 0.5 µm length scale for the bilayer)^40^, we have developed a two-dimensional model in which each copy of a membrane protein is represented by a single particle moving in the plane of the membrane (see SI for a more detailed description of the mesoscale model). To parameterise the dynamics of this model we have measured translational and rotational motions of OMPs as a function of cluster size in the CG–MD simulations (see Fig. 5a, b). Both translation (in the bilayer plane) and rotation (about the centre of mass of each cluster, in the plane of the bilayer; both over a period of e.g. 10 ns) depend strongly on cluster size. To extrapolate to the larger clusters which we anticipate in the mesoscale simulation sizes, we fitted observed RMS rotation or translation vs. cluster size data using a power law function (see Supplementary Table 1 for further details).

As has been observed previously^2,8^, interactions between the OMP molecules in clusters occur via well-defined interfaces (see Supplementary Figure 4). To incorporate this feature within our two-dimensional mesoscale model, we analysed the density of occurrence of adjacent proteins around a central BtuB or OmpF molecule, averaged across all such molecules and the duration of a CG–MD simulation (see Fig. 5c and Supplementary Figure 8). This analysis revealed that BtuB interacts principally via two interfaces whereas there are 3 main interaction areas for the trimeric OmpF molecule (Supplementary Figure 8A). In the mesoscale model these interfaces were represented as sticky patches of the protein particles. These areas of interaction influence the topology of clusters for the two different systems. Thus, clusters of OmpF proteins are more branched than clusters containing only BtuB proteins (see Fig. 1 and Supplementary Figure 2).

Thus, the dynamic behaviours of the protein particles in the mesoscale model are parametrised directly from the corresponding CG–MD simulations. In the mesoscale model, at each timestep each protein undergoes a translation and a rotation, selected from the CG–MD-derived distributions as described above. If two protein molecules encounter one another through their sticky patches they form a dimer. Once a dimer is formed it moves as a single entity, with translations and rotations selected from the CG–MD distributions for dimers. As successive proteins interact, larger clusters form and each cluster subsequently moves as a single entity, again with its motion determined from the CG–MD distributions. From our fitting onto the CG–MD simulations, we defined a single step in the mesoscale model as 10 ns in the CG–MD simulation timescale.

### Clustering and dynamics of OMPs at the mesoscale

**Fig. 6:**
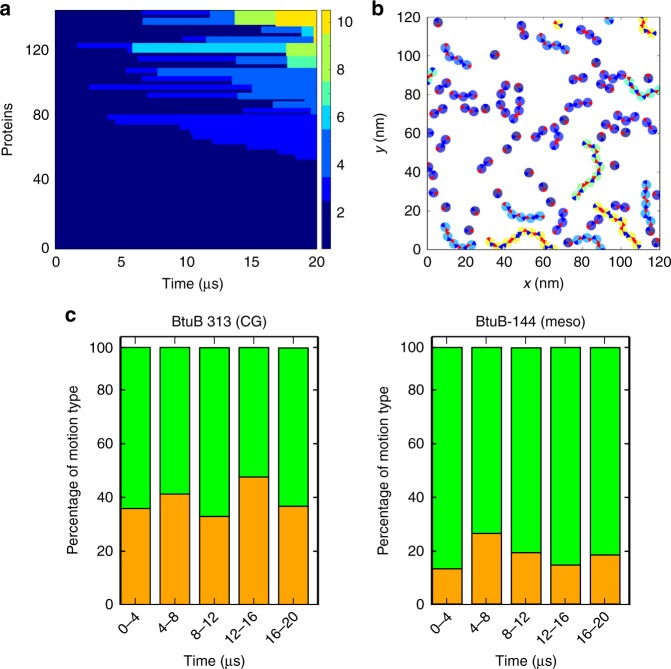
Mesoscale simulation of a BtuB-containing membrane. **a** Clustering as a function of time in a mesoscale simulation of 144 BtuB molecules (at 313 K) for 20 µs. Cluster size is shown by the colour bar. **b** Snapshot of the same simulation as in **a** at 20 µs. Proteins are coloured by cluster size, as in **a**. Protein interactions interfaces are shown as blue and red wedges on each protein. **c** Fraction of different classes of diffusional motion for equivalent coarse-grained (left) and mesoscale (right) simulations (as analysed using PaTrack^39^; see Fig. 3) over 20 µs of each simulation. Motions are classified as Brownian (green), or mixed Brownian and confined (orange)

We evaluated the mesoscale model by a simulation comparable to our CG–MD simulations of BtuB, i.e. a 20 µs mesoscale simulation of 144 BtuB molecules in a 120 × 120 nm^2^ membrane (Fig. 6). The extents of the sticky patches and other parameters of the mesoscale simulation were checked by comparison with the outputs of the CG–MD simulations (Fig. 6, and Supplementary Figures 9, 10). In particular, the clustering behaviour of the mesoscale model was seen to reproduce that of the CG–MD simulation, such that after 20 µs both monomeric and clustered proteins were present, with the largest clusters containing 10 or more proteins (Fig. 6a, b). By inspection, the clusters were generally linear rather than branched, again as observed in the CG–MD simulations. In terms of dynamic behaviour, after 20 µs of simulation, 20–40 proteins exhibited mixed motions while the remaining protein motions were Brownian, again in broad agreement with the CG–MD simulations (Fig. 6c and Supplementary Movie 2). Thus, the mesoscale model seems to capture the essential behaviour of the underlying CG–MD simulations. In terms of computational performance, at the CG–MD level this simulation required ~410,000 cpu.h for 20 µs, whereas the mesoscale simulation required <1 cpu.h. Thus, the mesoscale model enables much larger and longer simulations, permitting direct comparison with experimental observations via single molecule tracking.

**Fig. 7:**
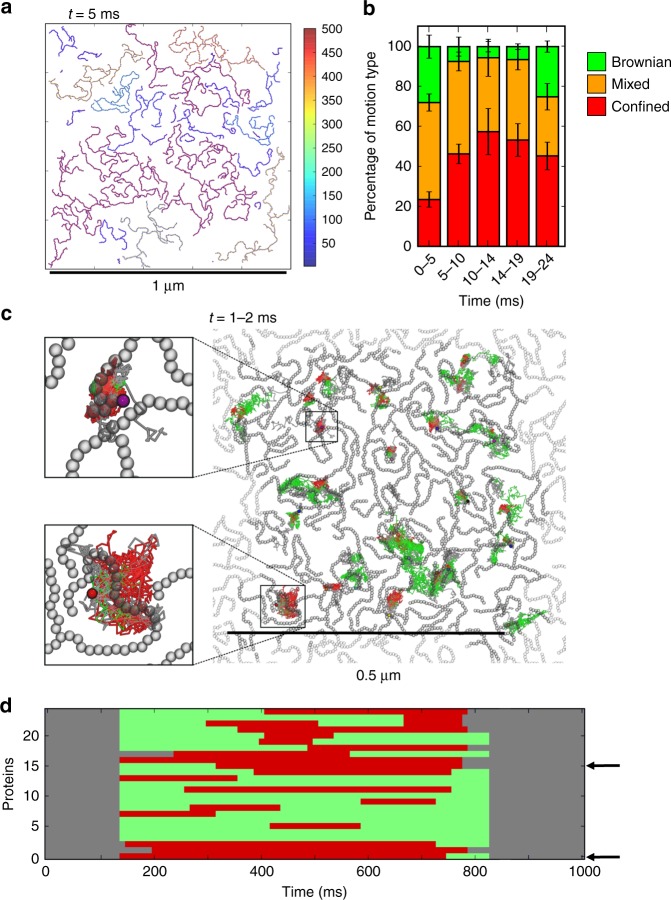
Mesoscale simulations at experimental scales. **a** Snapshot of a 24 ms duration mesoscale simulation of a patch of membrane containing 4900 proteins in a 1 µm^2^ box at 5 ms. The patch dimensions correspond to those of an experimentally observed OMP island. The colour scale represents protein cluster size. **b** Fraction of different classes of diffusional motion (Brownian, green; confined, red; or mixed Brownian and confined, orange) for a random sample of 100 from the 4900 proteins in the mesoscale simulation as a function of time. Error bars show the standard deviation given by taking five repeat resamples of 100 proteins. **c** Tracking over 1 ms of 25 inserted proteins. These were added to the mesoscale simulation in **a** after 1 ms, which was then allowed to run for a further 1 ms. The traces for the inserted proteins are coloured according to the motion type, as in **b**. The snapshot is from the final frame (i.e. at 2 ms) with the 2500 original proteins in grey, and their periodic repeats in pale grey. The trajectories of the newly inserted proteins are shown in red (confined) or green (Brownian). The upper inset shows a protein, which becomes part of a larger cluster and displays confined motion whilst the lower inset shows a protein that remains monomeric but exhibits confined motion as a result of being corralled by the pre-existing (in grey) cluster of proteins. **d** Classification of the diffusional motion of the 25 inserted proteins over the 1 ms simulation following their insertion. Colouring is as in **c**. (The grey blocks at either end of the trajectories are because the classifications employ a sliding window, and therefore these points are unclassified.) Arrows indicate the two protein trajectories displayed in the insets in **c**

We then used the mesoscale model to perform a long timescale (24 ms) simulation of a large patch (0.5 µm^2^) containing 4900 copies of the BtuB protein molecule in a 1 µm^2^ box (Fig. 7a; Table 1, simulation Meso: BtuB_interfaces). As observed in the CG–MD simulations, the clusters are heterogeneous with a large fraction of extended clusters containing more than 150 protein molecules. Thus, we see single clusters of dimensions comparable to the 0.5 µm length scale observed experimentally for OMP islands. Smaller clusters were also observed, often formed by fewer proteins intercalated in between these more extended clusters. In terms of dynamic behaviour, the proportion of mixed and confined protein diffusion trajectories increased over time and subsequently decreases slightly (Fig. 7b), reaching values comparable to in vitro experimental results (Fig. 3b). Furthermore, the diffusion coefficients for BtuB molecules in both the Brownian and the confined regimes of the 24 ms mesoscale simulation (e.g. 0.4 µm^2^ s^−1^ for Brownian diffusion) approached the diffusion coefficient of BtuB molecules observed experimentally (0.02–0.4 µm^2^ s^−1^ for Brownian diffusion; Supplementary Table 2 and Supplementary Figure 5B). Additionally, the spatial extent of the 24 ms mesoscale simulation trajectories is in the same range as in vitro observations (Supplementary Figure 11). The extended clusters exhibit occasional branching. These extended clusters may create moving corrals which can sequester smaller clusters in localised areas (see Supplementary Movie 3). Within a bacterial OM, this would create areas of restricted diffusion within which corralled membrane proteins would be expected to have a higher likelihood of interaction with one another. Thus, such behaviour is anticipated to occur within the OMP ‘islands’ observed experimentally.

To explore the effect of nanoscale protein–protein interactions on cluster formation and the resultant corralling behaviour, we simulated a control simulation of a second patch of 4900 proteins in a 1 µm^2^ box for 24 ms, with the specific interaction interfaces removed, such that proteins within the cutoff distance can associate with one another regardless of their relative orientation (Table 1, simulation Meso: No_specific_interfaces). Within 1 ms, a single large cluster formed, which was more branched and more compact than the mesoscale BtuB_interfaces simulation (Supplementary Figure 12A and Supplementary Movie 4). Notably in this case, no small protein clusters become corralled by larger clusters since, given that there are no specific interfaces, all proteins that come into contact with one another become part of a cluster. The difference in diffusive behaviour between the BtuB_interfaces and No_specific_interfaces simulations is evident in the PaTrack analysis of motion type (Fig. 7b vs. Supplementary Figure 12B). Over the course of 24 ms, the No_specific_interfaces simulation exhibited predominantly mixed and Brownian trajectories and, after the first 5 ms, no confined trajectories were seen (Supplementary Figure 12B). Over the course of the simulation there is significant variation in the motion type observed (Supplementary Figure 12B), which is likely due to the stochastic variation in motion when observing the single large protein cluster.

To further explore the possible functional consequences of such corralling within an extended network of OMP clusters, we performed a mesoscale simulation designed to mimic the behaviour of proteins newly inserted by the BAM complex^41^. We first took a patch (0.5 µm^2^) containing 2500 BtuB molecules and simulated for 1 ms (simulation Meso: BtuB_1ms; Table 1 and Supplementary Movie 5). Next, 25 new OMP molecules were inserted into the resultant configuration, at positions distributed across the membrane system. We then extended the simulation of the new system for a further 1 ms and tracked the trajectories of each of the 25 newly inserted proteins (Fig. 7c, d, simulation Meso: Insert_25; Table 1). Many of these newly inserted proteins quickly displayed confined diffusion. This confined movement can partly be explained by the integration of the protein into larger clusters, which are themselves corralled by neighbouring clusters (see particles in the inset on Fig. 7c and Supplementary Movie 6). Interestingly, the protein shown in the lower inset in Fig. 7c was not incorporated into a cluster but rather was corralled by an extended cluster around it. The diffusive movement of this protein was still classified as confined. Thus, confined diffusive motion may arise not only by direct incorporation into an adjacent cluster^8^ but can also arise by corralling by the surrounding extended and slowly moving clusters. This may explain the heterogeneity of diffusive behaviour reported in crowded membranes which may confer a glasslike behaviour on such membranes^42^. Such glassy dynamics of crowded membrane proteins is likely to play a key role in the function of membranes in cells.

### Methodological reflections

It is useful to reflect on the likely limitation of the mesoscale model. In the current version of this model, when two protein molecules encounter one another via their respective ‘sticky patches’, they remain associated for the remainder of the simulation (i.e. they remain associated on a timescale of up to 25 ms). We do not know the *k*_off_ for such interactions, but we can provide order of magnitude estimates. From simulations of the coarse-grained free energy landscape of association of two OMPs^6^ we can estimate a free energy of association of ca. −50 kJ mol^−1^, corresponding to a *K*_d_ of the order of 1 nM. We can assume association rates to be in the range *k*_on_ = 10^6^–10^9^ M^−1^ s^−1^^43^, thus yielding a range of *k*_off_ = 10^−3^ to 1 s^−1^. This is consistent with dissociation timescales observed in high-speed AFM of OmpF^2^ and also with the diffusive behaviour of BtuB in vitro (Supplementary Figure 5A), which shows diffusion slowing on a minute timescale. Thus, the timescale on which we would expect to observe dissociation of OMP monomers from a cluster is likely to be seconds or greater.

There remain some differences between model and simulation, e.g. in the exact values of diffusion coefficients. It is also apparent that in the 24 ms Meso: BtuB_interfaces simulation there is a slight decrease in the level of confined motion in the last 5–10 ms of the mesoscale simulation, even though it might be expected that the level of confined motion should continue to increase over time. This is due in part to a concentration/dilution effect, as the proteins do not retain a microscopically homogenous distribution within the membrane with time, and additionally, as one or two large clusters form, there is no longer the same level of corralling of protein clusters by surrounding clusters and the motion becomes Brownian, albeit much slower Brownian motion (see also Supplementary Figure 5). However, we do obtain a semi-quantitative agreement with experiment, both in terms of diffusion modes and coefficients. An improved quantitative fit between simulation and experiment would likely require a more complex (and hence computationally demanding) modelling strategy which would then make it difficult to approach a match in experimental and simulated timescales and so prevent a direct comparison.

In terms of the system sizes for our mesoscale simulations, we have used ca. 1 µm patches to emulate the lengthscale of the in vitro measurements. In future studies we could extend our model to characterise the behaviour proteins on the full surface of an *E. coli* cell, by increasing the number of proteins simulated, by allowing for dissociation of monomers from a cluster, by incorporating full lipid complexity, and by including appropriate curvature to the surface.

### Biological implications

In simulating a 1 µm patch we provide a highly simplified model of a portion of the *E. coli* outer membrane. To what extent do these simulations inform our understanding of the dynamic organisation of bacterial OMPs? Much recent work has focussed on modelling the interactions of LPS in such membranes^44–46^. LPS was not included in our simulations, as we wished to mimic the simpler in vitro system discussed above. As discussed above, in *E. coli* outer membranes, estimates suggest that LPS is present at concentrations approaching those of OMPs, with the consequence that not all OMPs will be entirely encircled by LPS^28,47^. Taken alongside the evidence that LPS self-associates^48^ it is therefore likely that the outer membrane of *E. coli* is highly heterogeneous in terms of LPS density. Hence, defining self-association mechanisms of OMPs within a membrane devoid of LPS is likely to reflect interactions within highly dense OMP populations. This in turn is consistent with previous results showing that in vivo and in vitro systems show comparable behaviour^8^, and with the results presented in Supplementary Figure 1 showing that diffusion and clustering of OMPs is unaffected by the presence or absence of exogenously added LPS. Thus, the simulations done in the absence of LPS molecules may reflect the patchy nature of the OM and the presence of OMP-rich regions. Moreover, simulating OMPs in a lipid mixture identical to the in vitro system allows for an understanding of the relationship between molecular interactions and emergent mesoscale behaviour in the comparatively simple and well-defined in vitro reconstituted model. Thus, it is demonstrated that protein–protein interactions alone allow for an explanation the restricted motion observed in simple in vitro membranes, which in turn appears to explain the formation of OMP islands. Nevertheless, a model of the entire *E. coli* outer membrane will require incorporation of LPS, as well as, for instance, outer/inner membrane connections^12^. Having established the methodology, we will be able to build on the current mesoscale model.

## Discussion

We have developed mesoscale models of membrane protein dynamics, based on underlying CG–MD simulations, which have enabled us to probe the dynamic organisation of models of bacterial outer membranes on length and timescales approaching those observed experimentally. This enables us to provide a nanoscale molecular mechanism for the mesoscale in vitro observations. Our simulations suggest that the confined diffusive motion of bacterial OMPs observed experimentally^8^ corresponds to a mixture of extended protein clusters, an emergent property on the mesoscale of specific protein–protein interaction interfaces observed at the nanoscale, and of individual membrane proteins corralled by these clusters. This agrees well with recent measurement of the glasslike behaviour of crowded membranes^42^. Such corralling is likely to be of importance for OMPs newly inserted by the BAM machinery. More generally, this multiscale simulation approach will enable exploration of the molecular origins of clustering of signalling molecules in both bacterial (e.g. ref. ^49^) and mammalian (e.g. refs. ^50,51^) cell membranes.

## Methods

### In vitro single molecule tracking and co-localisation

Standard microscopy glass cover slides were functionalized with a high density poly(ethylene)glycol (PEG) brush with terminal palmitate as reported previously^52^. Proteoliposomes were formed from mixing both *E. coli* polar lipids (Avanti Polar Lipids) and OMPs solubilized in detergent with protein to lipid ratios previously described^8^, followed by vesicle formation by rapid detergent extraction using ß-cyclodextrin^52^. For additional controls, LPS was added to the lipid mixture to yield a 17:1 ratio of LPS:BtuB, as estimated from the fluorescence intensity of BtuB-ColE9-TMR and of fluorescent LPS-bodipy.

The proteoliposomes were incubated with the functionalized surface for 30 min, before membrane fusion was induced by 15 min of incubation with soluble PEG_8000_ in buffer, followed by removal of excess lipid material.

OmpF with a single point mutation (E29C) was site-specifically labelled with maleimide-Dy647 prior to the PSM formation, while BtuB was stained by 5 min of incubation with 10 nM of tetramethylrhodamine (or AlexaFluor488) labelled colicin E9 after formation of the PSM. (^TMR^ColE9 and ^AF488^ColE9; 97% and 98% of labelling according to absorbance analyses at 280 nm compared to 488 and 545 nm, respectively.) We estimate that less than 10% of BtuB molecules are linked to a fluorescent ColE9 molecule, based on the initial vs. final intensity comparisons at different concentrations of labelled ColE9. Simultaneous single molecule total internal reflection fluorescence microscopy (TIRFM) of different fluorescence channels was carried out as previously described^8,53^. After localising single point emitters in each channel by the multi target tracking algorithm^54^, an in-house routine was subjected to identify OMPs co-localising with an upper threshold of 200 nm (~2 pixels).

To estimate the fraction of the membrane surface area occupied by protein we first measured the average intensity of single BtuB-ColE9-TMR (i.e. one step photobleaching) when incorporated into a PSM at very low dilution. We then summed the intensities of pixels within several regions of interest (ROI) for a PSM incorporating BtuB at high concentration. Knowing the surface of each ROI, the intensity value corresponding to a single BtuB, and the transversal surface of a BtuB molecule, we can therefore calculate the surface coverage of BtuB over the PSM, yielding a figure from 20 to 40% from one experiment/ROI to another, with an average close to 25%.

TIRFM videos and image sequences from MD simulations were analysed using custom software (PaTrack)^39^. The centre of each fluorescence peak was determined with sub-pixel resolution by fitting a two-dimensional elliptical Gaussian function. The two-dimensional trajectories of single molecules were constructed frame-by-frame, selecting particles that displayed a single bleaching step. Diffusion coefficient values were determined from a linear fit to the MSD plots between the first and fourth points according to the equation: MSD(*t*) *=* 4*Dt*.

### Coarse-grained molecular dynamics

Simulation set-up: Three pairs of CG–MD simulations were performed: simulations containing 144 BtuB molecules; simulations containing 72 BtuB and 72 OmpF trimers; and simulations containing 100 OmpF trimers, all of which had dimensions of roughly 140 × 140 × 15 nm^3^. In each case, two 20 µs simulations were performed, a first at 313 K and a second at 323 K. One very large system, a membrane patch of 480 × 480 nm^2^, containing 1152 ButB and 1152 OmpF at 323 K, was simulated for 2.5 µs.

Structures of BtuB and OmpF (PDB ID: 2YSU [dx.doi.org/10.2210/pdb2YSU/pdb] and 2OMF [dx.doi.org/10.2210/pdb2OMF/pdb]) were converted to a CG representation using the martinize.py script (version 2.4) downloaded from the MARTINI website (http://md.chem.rug.nl/index.php/tools2/proteins-and-bilayers) with the MARTINI2.2 force field^34,55^ and the ElNeDyn elastic network model^56^. The BtuB structure, 2YSU, had missing loop residues, which were modelled using Modeller^57^.

A grid of randomly rotated proteins was embedded into a pre-equilibrated phosphatidyl choline (PC) bilayer using g_membed^58^. For simulations including BtuB or a mix of BtuB and OmpF, this initial grid contained 6 × 6 proteins; for simulations containing OmpF the initial grid contained 5 × 5 OmpF trimers, to take account of the difference in size between BtuB and the OmpF trimer. Using the exchange lipid protocol^59^, the lipid composition was swapped from all PC to PE and PG in a 3:1 ratio, chosen since it mimics the composition of the in vitro membrane. Water and neutralising counterions were added. After a short (100 ns) equilibration with proteins restrained, the (6 × 6 or 5 × 5) systems were tessellated, using the Gromacs tool genconf, to make larger systems containing 12 × 12 or 10 × 10 proteins, approximately 25,000 lipids and approximately 800,000 waters. A further very large system was constructed by tessellating the 6 × 6 BtuB–OmpF mixture to create a system containing 48 × 48 proteins (1152 BtuB monomers and 1152 OmpF trimers).

Simulation parameters: All simulations were performed using GROMACS 4.6^60^ (www.gromacs.org) and the standard MARTINI protocol^33,34^. Periodic boundary conditions were applied, and a time step of 20 fs was used in all simulations. The temperature was maintained at either 313 or 323 K using a Berendsen thermostat^61^, and the pressure at 1 bar using a Berendsen barostat. For both the temperature and pressure, a coupling constant of 4 ps was used for all simulations. The reaction field coulomb type was used with a switching function from 0.0 to 1.2 nm, and the van der Waals interactions were cutoff at 1.2 nm with a switching function applied from 0.9 nm. The LINCS algorithm^62^ was used to constrain covalent bonds to their equilibrium values. All systems containing protein were simulated for 20 μs, except for the very large system (48 × 48 proteins) which was simulated for 2.5 µs.

### Mesoscale model

In the mesoscale simulations, each protein is considered as a single particle. At each time step, proteins are translated and rotated by an amount sampled from distributions of rotations and translations, which are themselves obtained from CG–MD simulations. At each step, proteins that are considered to be interacting (via specific interaction interfaces as determined from CG–MD simulations), are clustered. Thus protein clusters are formed and develop.

The distributions governing protein motions are dependent on protein cluster size (defined as the number of proteins in a given cluster) and are taken from the CG–MD simulations (Fig. 5a, b). Protein coordinates are restricted to two dimensions, that is, the assumption is that the membrane is flat. In mesoscale simulations of 24 ms duration containing 4900 proteins (BtuB_interfaces and No_specific_interfaces; see Table 1) proteins were placed in a box larger than the size of the initial protein patch, and the edges of the box acted as a hard boundary, as in a PSM. In terms of the mesoscale model, this means that if the movement of a protein would cause it to move outside the box area, that move is rejected. Periodic boundary conditions were implemented for mesoscale simulations: BtuB_1ms and Insert_25 in order to mimic the centre of a protein island.

Starting configuration: In all mesoscale simulations, a starting conformation consistent with the CG–MD simulations was conserved. Thus, proteins were positioned on a square grid, at a distance of 10 nm from horizontal and vertical neighbours. In simulations with 4900 proteins, the initial protein patch encompassing 700 nm × 700 nm was placed in the centre of a 1 µm^2^ box. In simulations with 2500 BtuB proteins, the protein grid covered the simulation box, such that the box had dimensions 500 nm × 500 nm.

Protein–protein interactions: Protein–protein interactions in the mesoscale model are controlled by three parameters. The first is the protein diameter, modelled for BtuB as 5 nm; proteins within 5 nm of one another are considered as possibly interacting. The second protein–protein interaction parameter is an overlap margin value, which defines the minimum distance between any two proteins. The third is the size and location of so-called ‘sticky patches’, which were defined according to the CG protein interaction density (Fig. 5c).

Thus proteins within a cutoff of 5 nm (and above a cutoff distance of 4.9 nm) of one another are considered to be interacting only if facing each other via ‘sticky patches’, that is, such that arctan((*y*_2_ − *y*_1_), (*x*_2_ − *x*_1_)) − *θ*_1_ and arctan((*y*_2_ − *y*_1_), (*x*_2_ − *x*_1_)) + *π* − *θ*_2_ lie in a specific range, where *x*_*i*_*, y*_*i*_ *and θ*_*i*_ are the *x*- and *y*-coordinates and orientation of the *i*-th protein. The angle ranges used for BtuB are 15–80° and 180–230°. These angles were chosen as shown in Fig. 5c and Supplementary Figures 9, 10. The ‘sticky patches’ as described above were used in all mesoscale simulations except the ‘No_specific_interfaces’ simulation (see Table 1), in which proteins within the cutoff of 5 nm (and above a cutoff distance of 4.9 nm) were considered to be interacting regardless of their relative orientation.

Interacting proteins form clusters, which are characterised by the proteins within a single cluster moving as one, that is, all proteins in a cluster are translated by the same amount and are rotated about the protein cluster centre of mass by the same angle. Proteins within 5 nm of one another but not interacting via their protein–protein interfaces continue to move independently of one another, but are sterically hindered—i.e. no pair of proteins can move within 98% of the 5 nm cutoff (i.e. 4.9 nm) of one another.

Update step: The system information that is stored between each update step is, for each protein, the *x*-coordinate, the *y*-coordinate, and *θ*, the orientation of each particle. Also stored is a list of clusters, containing the ID of each protein in a given cluster.

Each cluster is assigned a rotation and an *x*- and *y*-translation (see subsection below). Each cluster is then rotated about its centre of mass and translated. Pairwise distances are calculated. If any proteins are found to be ‘clashing’ (that is, the distance between proteins is less than 4.9 nm) then the move of the cluster that they belong to is rejected. If this move itself causes a clash with a further cluster, the third cluster is also moved back to its original position, and so on, until there are no clashes. Given that the translation and rotation steps are small, this does not affect many clusters.

Finally, after each cluster has been translated and rotated, all protein pairwise distances and orientations are used to determine whether proteins are interacting and therefore considered to be ‘clustered’.

Rotation and translation distributions: Rotations are picked from a Gaussian distribution of rotations, with mean zero and standard deviation as specified by CG–MD simulations. The standard deviation depends on the protein cluster size (Fig. 5a).

Translations are picked from a Gaussian distribution of translations, with mean 0 and standard deviation as defined from the CG–MD simulations (Fig. 5b). Translation in the *x*-direction and translation in *y*-direction are sampled separately, thus generating a direction and a magnitude for translation.

To extrapolate values for translation and rotation to clusters containing more than 20 proteins the standard deviation of the rotations and one-dimensional translation vs. cluster size data was fitted using a power law function, *y* *=* *Ax*^−*b*^, where *x* is the cluster size and *y* is the rotation or translation as appropriate. Residual values for the fitting are shown in Supplementary Table 1 and show that the power law fitting was appropriate.

The mesoscale simulations were performed using scripts written in Python, which made use of the NumPy^63^, MDAnalysis^64^ and NetworkX^65^ Python libraries.

### Analysis of simulation data

Analysis of CG–MD clustering and protein–protein interactions: Clustering analysis was performed using in-house Python scripts, making use of the NumPy, MDAnalysis and NetworkX Python libraries. In the clustering algorithm, proteins were considered to be interacting when the centroids were within 6 nm of one another (for BtuB–BtuB pairs), 8.8 nm (for pairs of OmpF trimes) and 7.3 nm (for a BtuB–OmpF pair). Protein–protein interactions were identified using in-house clustering scripts, again making use of the NumPy and MDAnalysis modules. Residues of neighbouring proteins were considered to be interacting when residue centroids were within 0.7 nm of one another.

Analysis of CG–MD and mesoscale simulations using PaTrack: In order to analyse the simulations using the single molecule tracking analysis package, PaTrack^39^, CG–MD and mesoscale simulations were first converted to videos with an in-house Python script that made use of the libraries NumPy and SciPy. The videos were converted to spe format using ImageJ^66^ (https://imagej.nih.gov/ij/index.html) and then analysed as for conventional single molecular tracking data using PaTrack.

Visualisation: Systems were visualised using VMD^67^ and graphs plotted using matplotlib^68^ (https://matplotlib.org).

### Data availability

Data supporting the findings of this manuscript are available from the corresponding authors upon reasonable request. The code used to run mesoscale simulations can be found at https://github.com/annaduncan/meso_model.

## Electronic supplementary material

Supplementary Information

Description of Additional Supplementary Files

Supplementary Movie 1

Supplementary Movie 2

Supplementary Movie 3

Supplementary Movie 4

Supplementary Movie 5

Supplementary Movie 6
