# Supplementary material for: How nanoscale protein interactions determine the mesoscale dynamic organisation of bacterial outer membrane proteins
Source: Nat Commun. 2018 Jul 20;9:2846. doi: 10.1038/s41467-018-05255-9 (PMC6054660; doi:10.1038/s41467-018-05255-9)
Supplement: Supplementary file 1 — Supplementary Information [file 41467_2018_5255_MOESM1_ESM.pdf]

Supporting Information for:

How Nanoscale Protein Interactions Determine the Mesoscale Dynamic Organisation of Bacterial Outer Membrane Proteins

Matthieu Chavent, Anna L. Duncan, Patrice Rassam, Oliver Birkholz, Jean Hélie, Tyler Reddy, Dmitry Beliaev, Ben Hambly, Jacob Piehler, Colin Kleanthous and Mark S. P. Sansom

Supplementary Figures

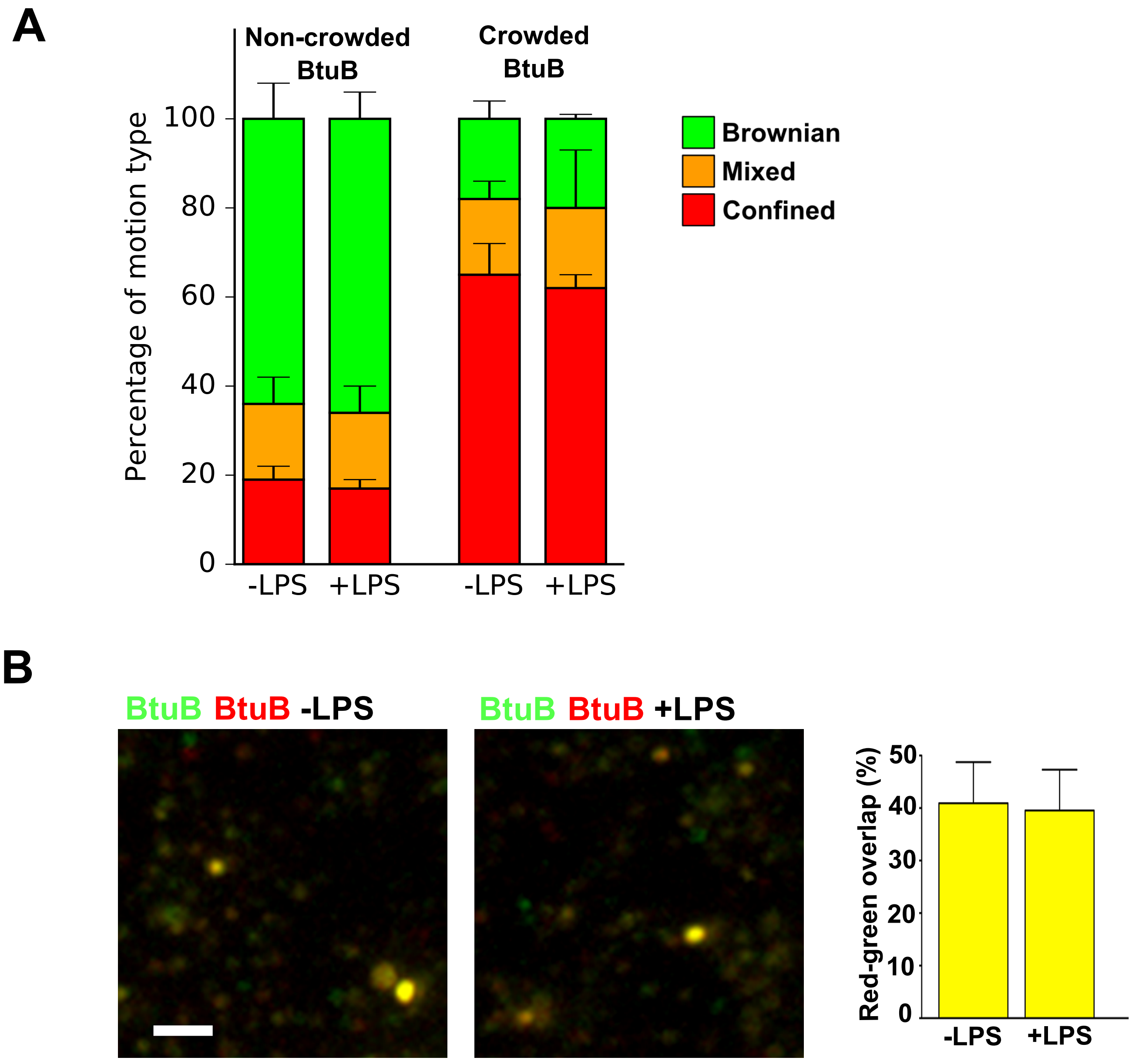

Supplementary Figure 1. In vitro single molecule tracking and colocalisation data showing that BtuB behaviour is not altered by the presence of LPS.

A. Proportion of tracked BtuB having Brownian (green), mixed (orange) or confined (red) motion, with and without LPS and for two degrees of BtuB crowding (raw data taken from Rassam *et al.*, Nature, 2015). Error bars show standard error of mean, n = 3. **B.** Images shows BtuB (green and red; overlap in yellow) in supported bilayers without (left) and with (centre) LPS (scale bar 1 µm). Bargraph (right) showing the extent of BtuB colocalisation (percentage of red-green overlap). Error bars show standard error of mean, n = 3 .

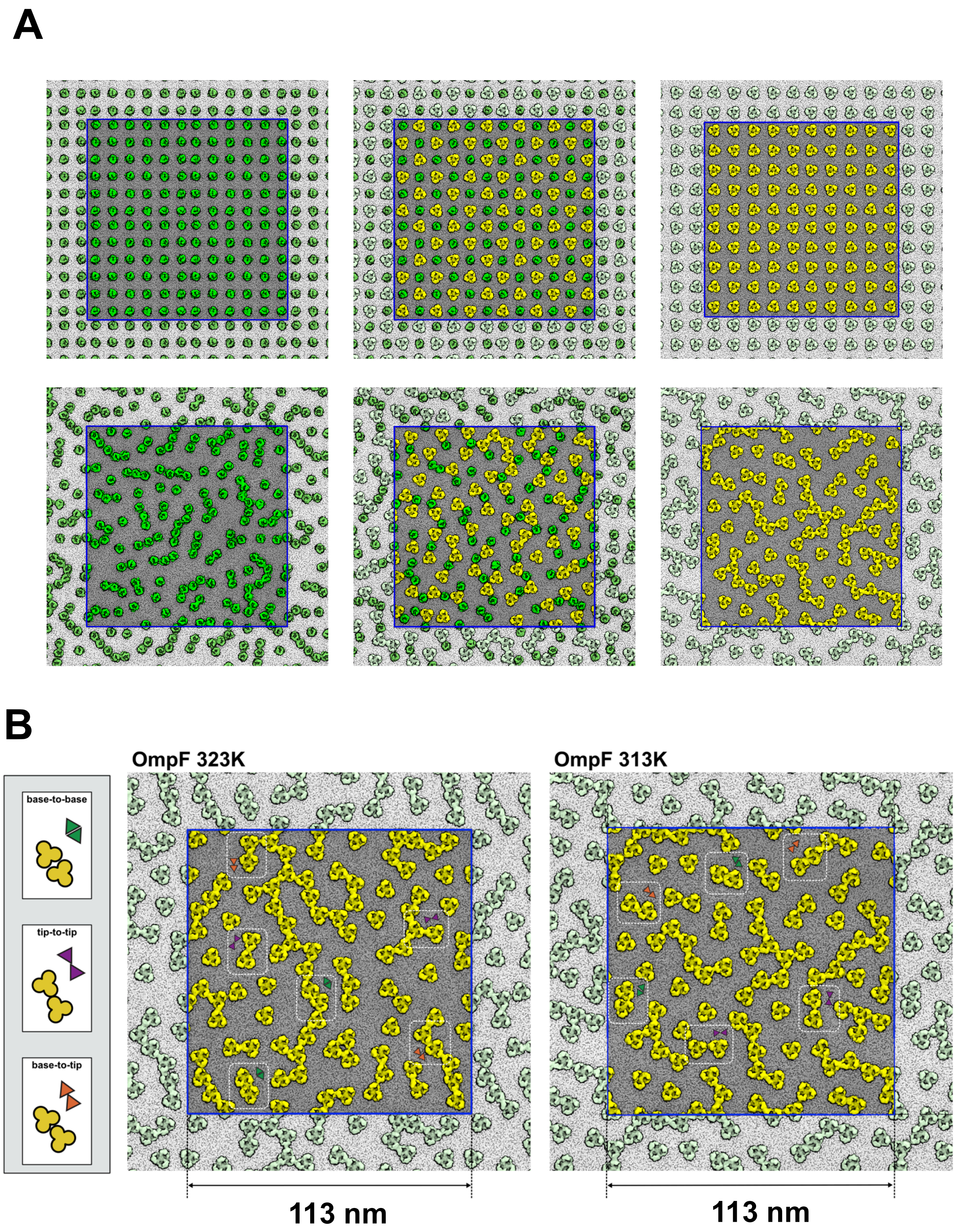

Supplementary Figure 2. Starting and final positions of systems.

A. Snapshots at 0 μs (upper row) and 20 μs (lower row) of the three different protein compositions (BtuB, left; OmpF, right; and mixed BtuB and OmpF, middle) embedded in a lipid bilayer (POPE:POPG 3:1) simulated at 313 K. Simulations of the systems at 323 K are shown in Figure 1 in the main text. **B**. Snapshot of OmpF simulations at 20 μs, demonstrating (left inset) the variety of orientations observed for OmpF-OmpF interactions. These interactions are equivalent with configurations based on AFM experiments. [1]

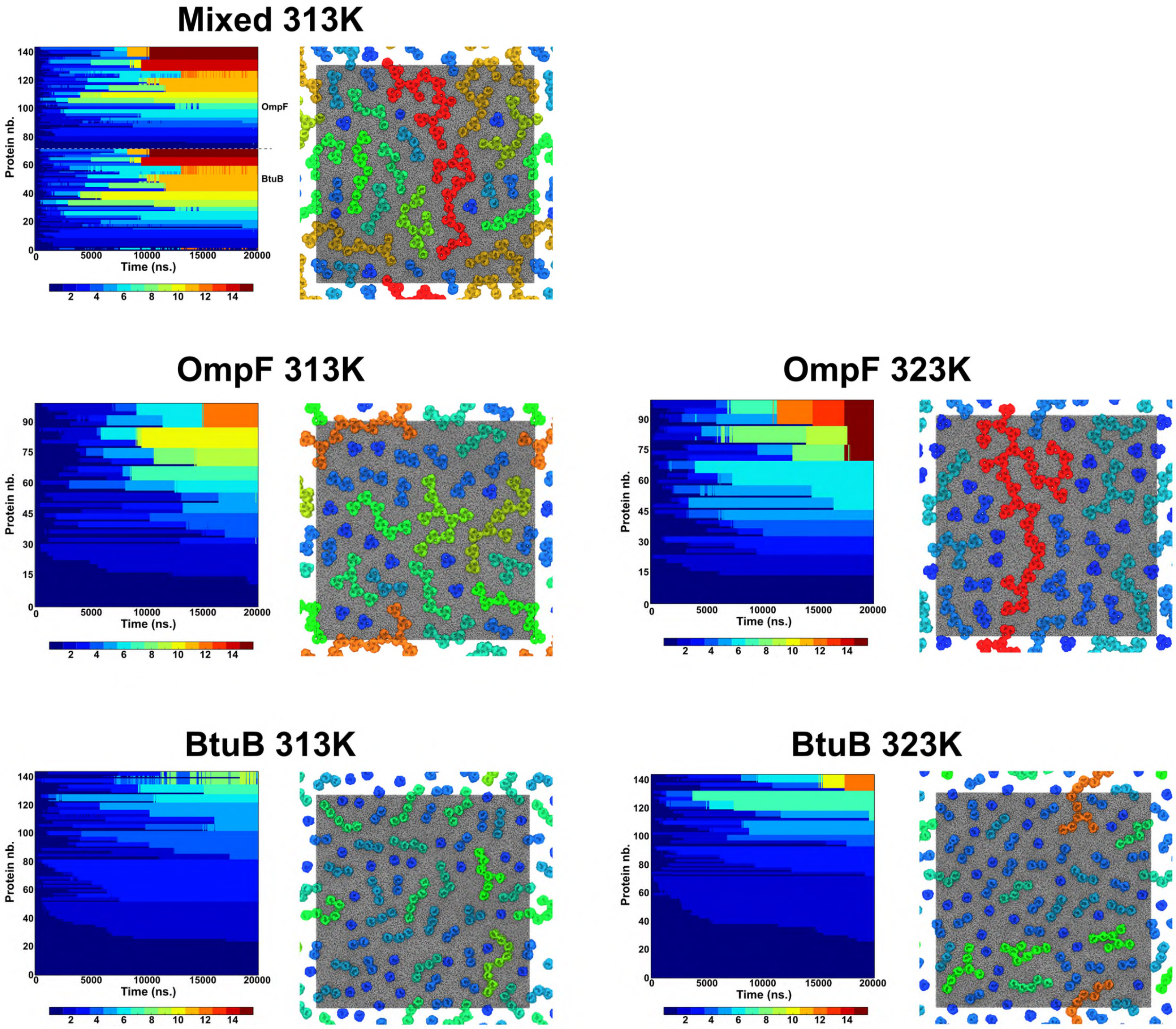

Supplementary Figure 3. OMPs clustering over time.

Nanoscale clustering of BtuB and OmpF for all simulations (except for Mixed323, which is shown in the main text in Figure 2). On the right of each panel is the final snapshot at 20 μs. On the left of each panel is the temporal evolution of cluster sizes over the 20 μs of each simulation. Proteins are coloured according to the cluster size of which they form a part (scale according to the colour bar).

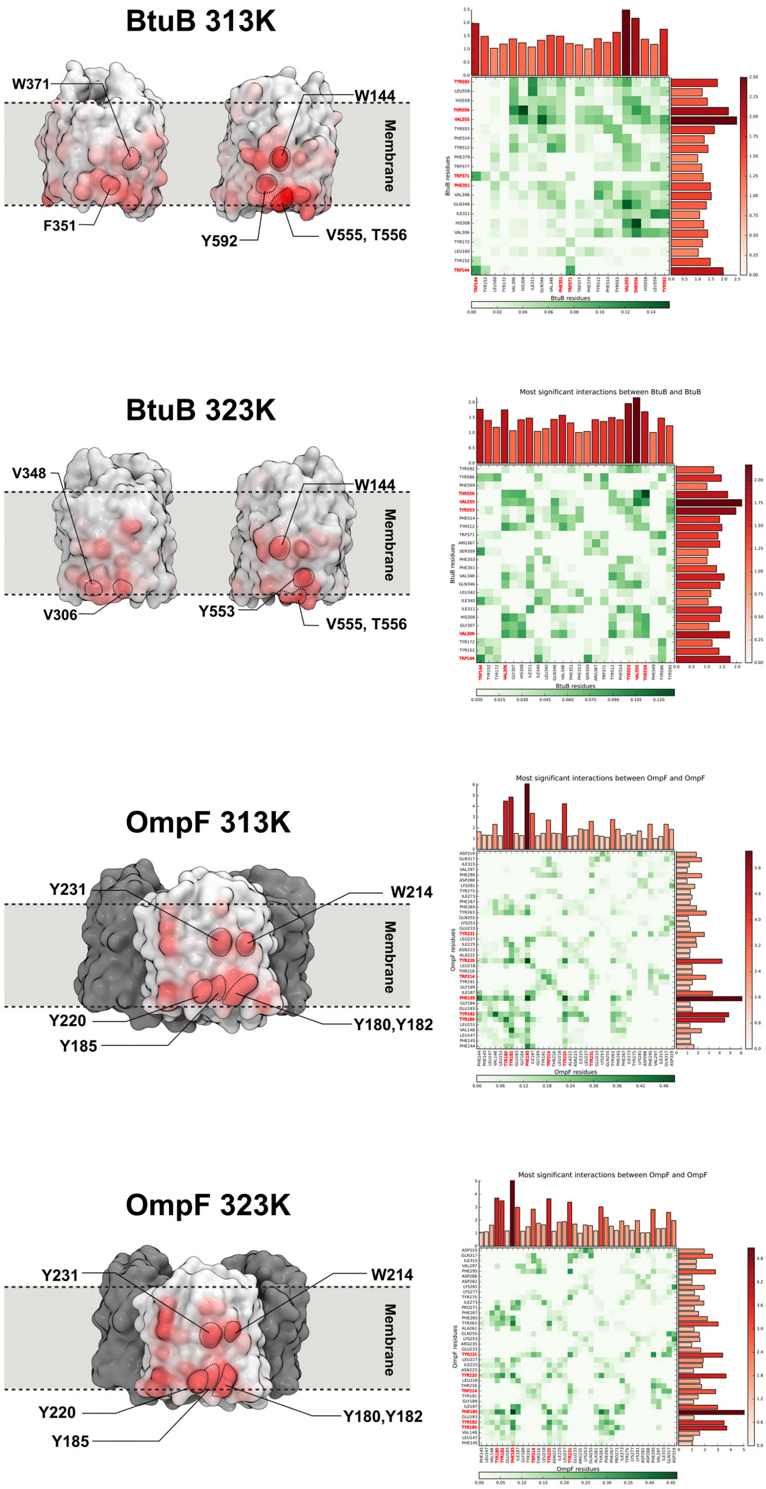

Supplementary Figure 4. BtuB and OmpF interfaces.

High frequency interaction sites for protein-protein interactions in each simulation, displayed on the protein structure (left) using a white (low) to red (high frequency of interaction) heatmap, and (right) as a protein-protein interaction matrix. For Btub-OmpF systems, residues in interactions are equivalent to ones seen in Rassam *et al.* [2] (data not shown). On the protein-protein interaction matrices (right column), residue pair interactions are shown as a percentage of the total residue pair interactions (white to green, scale shown on colour bar), and percentage of interactions by the each residue with all other residues on any neighbouring proteins is shown as a percentage of total residue interactions (white to red, scale shown on colour bar).

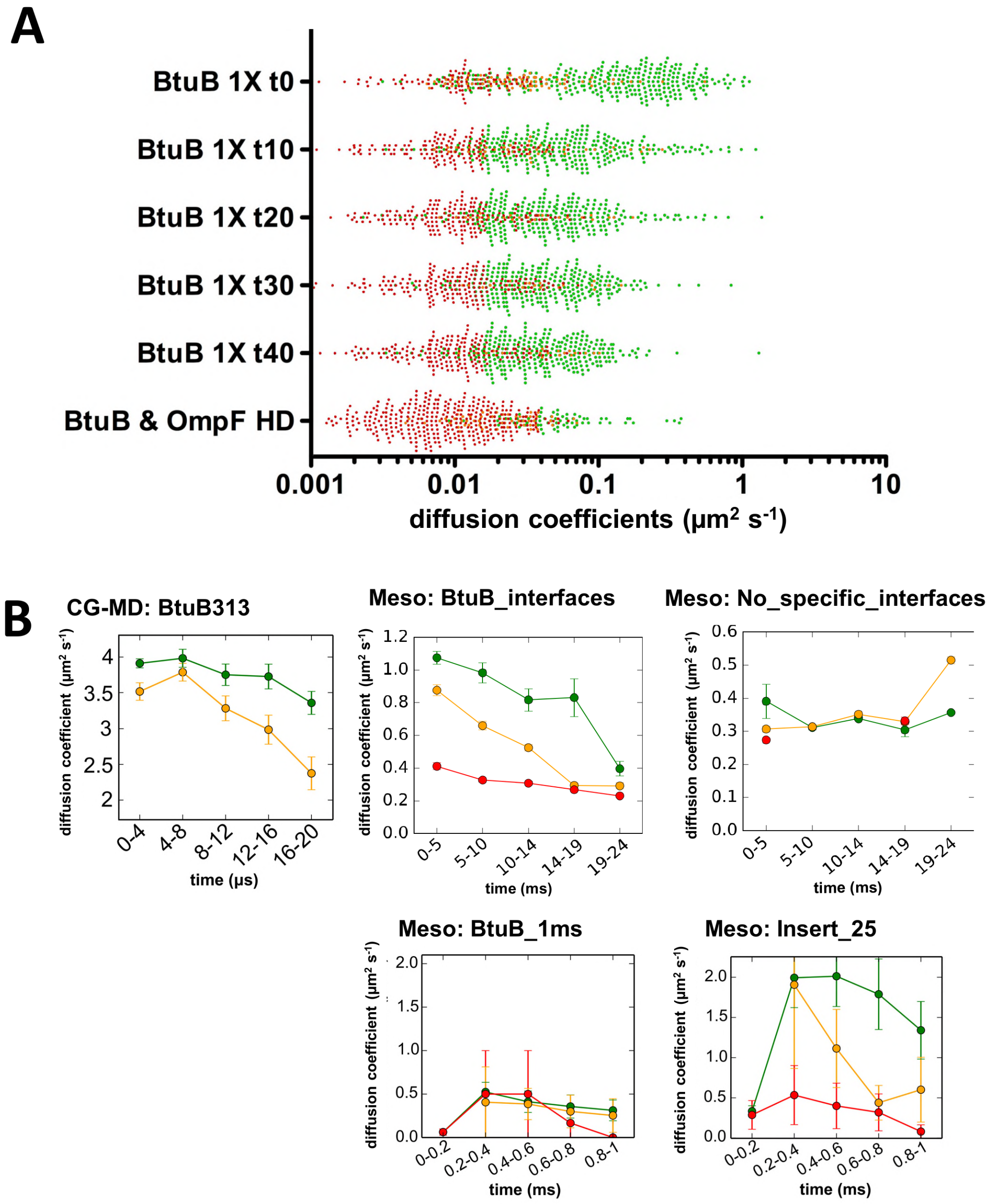

Supplementary Figure 5. In vitro and simulated diffusion.

A Diffusion coefficients of BtuB in vitro at t = 0,10, 20, 30, and 40 minutes after adding labelled ColE9 to the polymer supported membrane (top five rows) and of BtuB in the presence of a high density (HD) of OmpF (lower plot). **B** BtuB diffusion coefficients for: the CG-MD BtuB313 simulation (see Table 1), estimated over 4 µs time blocks of the simulation; for the Meso BtuB_interfaces simulation, averaged over 4.8 ms time blocks of the simulation; for the Meso No_specific_interfaces simulation, averaged over 4.8 ms time blocks of the simulation; for the Meso BtuB_1ms simulation, averaged over 0.2 ms time blocks of the simulation; and for the 25 newly inserted BtuB molecules in the Meso Insert_25 simulation, estimated over the 0.2 ms time blocks of the simulation. For the Meso:BtuB_interfaces and Meso:No_specific_interfaces simulations, which both contained 4900 proteins, the diffusion values shown are for five repeat analyses, each randomly sampling 100 proteins from the total 4900 proteins. The protein motions were classified as Brownian (green), confined (red), or mixed (yellow) using PaTrack and their diffusion coefficients averaged within each such group. In Meso: BtuB interfaces and Meso: No_specific_interfaces, error bars show standard deviation of all 500 (100 x 5) proteins. In the Meso: BtuB_1ms and Meso: Insert_25, error bars show standard deviation of all 100 proteins sampled for PaTrack analysis. Also see Supplementary Table 2, below.

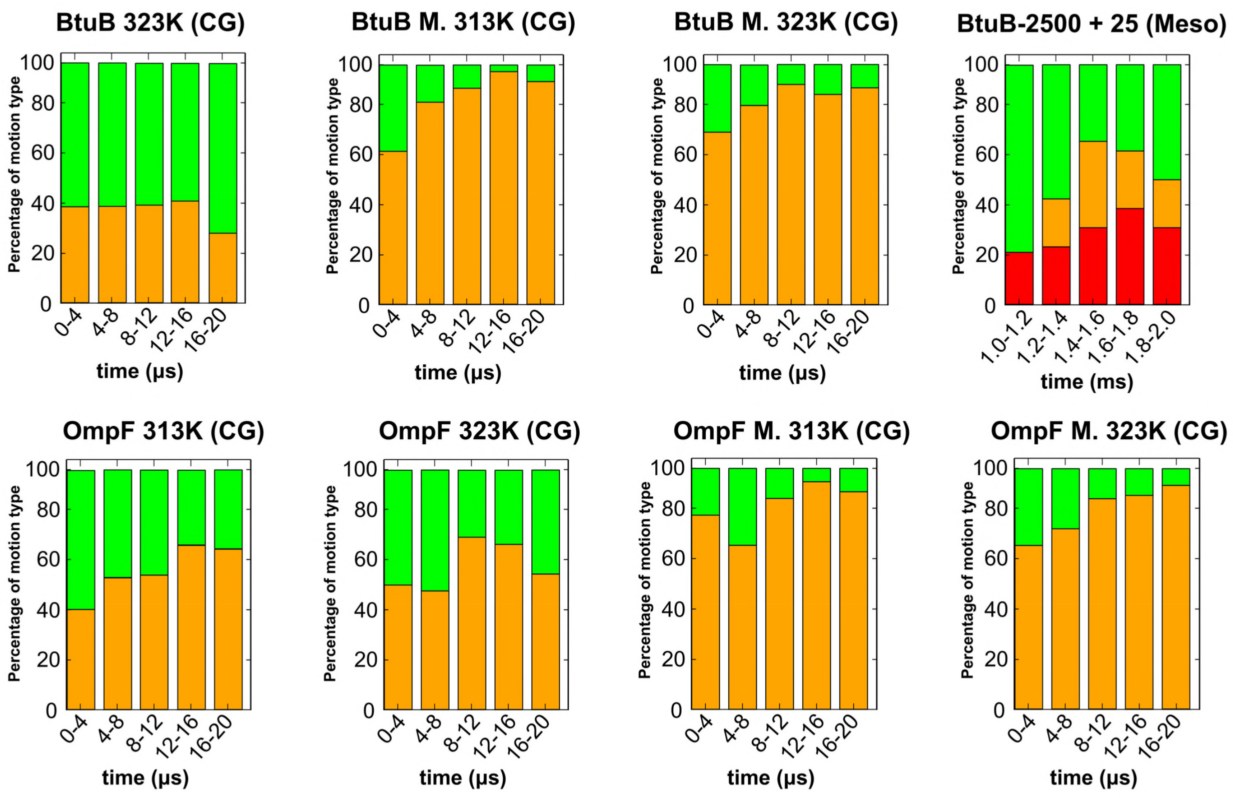

Supplementary Figure 6. In silico tracking

Analysis of in silico trajectories (CG-MD and mesoscale) using PaTrack [3], the tracking program developed to follow in vitro protein trajectories. PaTrack classifies trajectories into three type of motions: Brownian (green), confined (red), and mixed i.e. trajectories depicting both Brownian and confined movements (orange). The trajectories were divided in 5 equal segments to assess the evolution of the protein dynamical behaviour over time. For all the CG systems, we see a larger proportion of Brownian motion for BtuB than for OmpF proteins. This can be related to the respective sizes of the proteins as previously highlighted [4] . With the mesoscale model it is possible to reach time scales an order of magnitude longer than CG-MD simulations. This allows formation of larger clusters and the appearance of confined motion. This is in agreement with in vitro results.

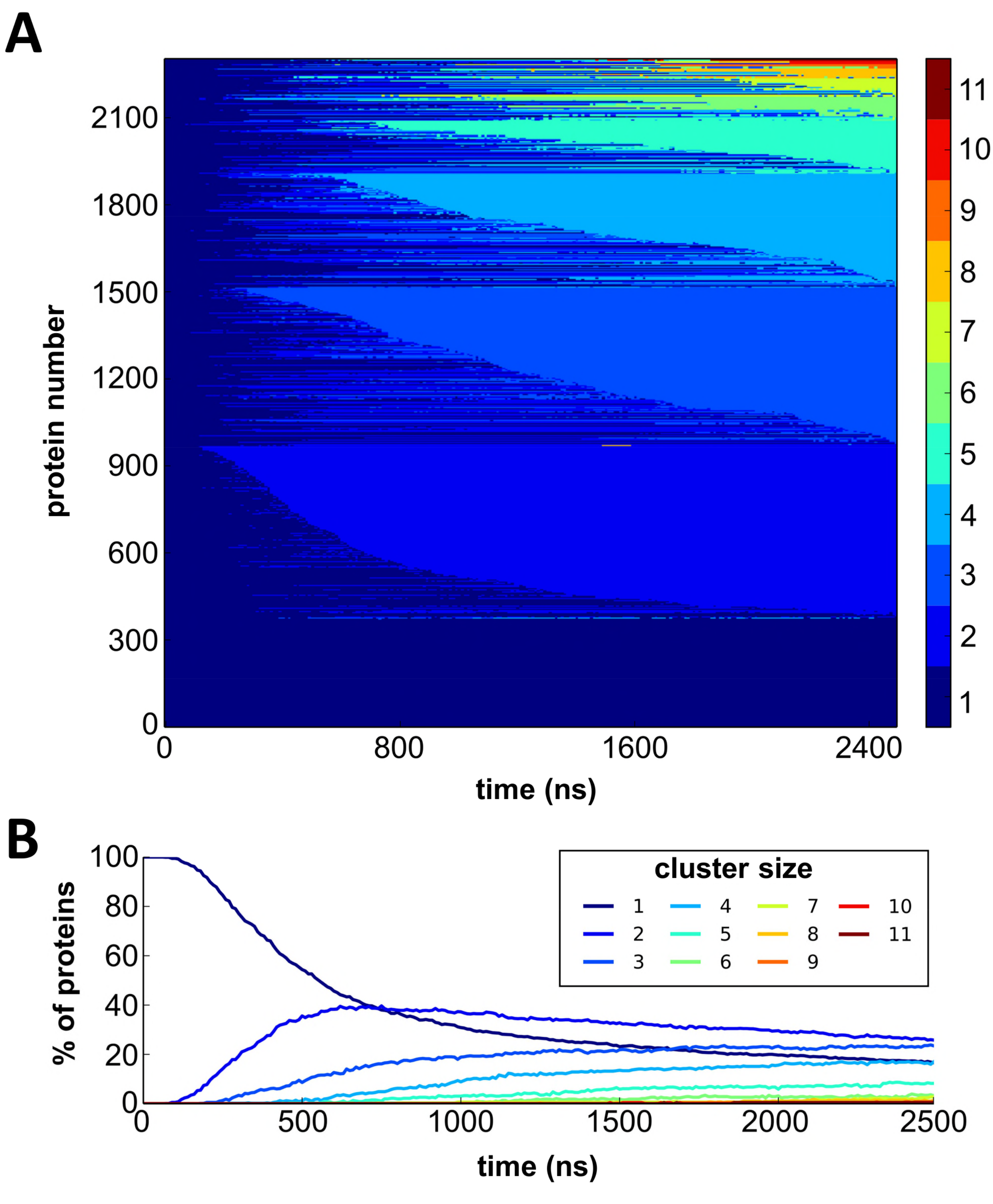

Supplementary Figure 7. Clustering of BtuB and OmpF for the 480x480 nm^2 system.

A Evolution of cluster sizes over the course of 2.5 µs for the vBig simulation (see Table 1). As seen for smaller systems, after 2.5 µs there is a formation of higher order clusters up to a size of 11 proteins in a cluster. **B** During the simulation, the monomer population quickly decreases and dimers are formed. Subsequently, trimers and higher order clusters up to a cluster size of 11 are formed. Proteins are coloured according to cluster size, as indicated on the colour scale bar.

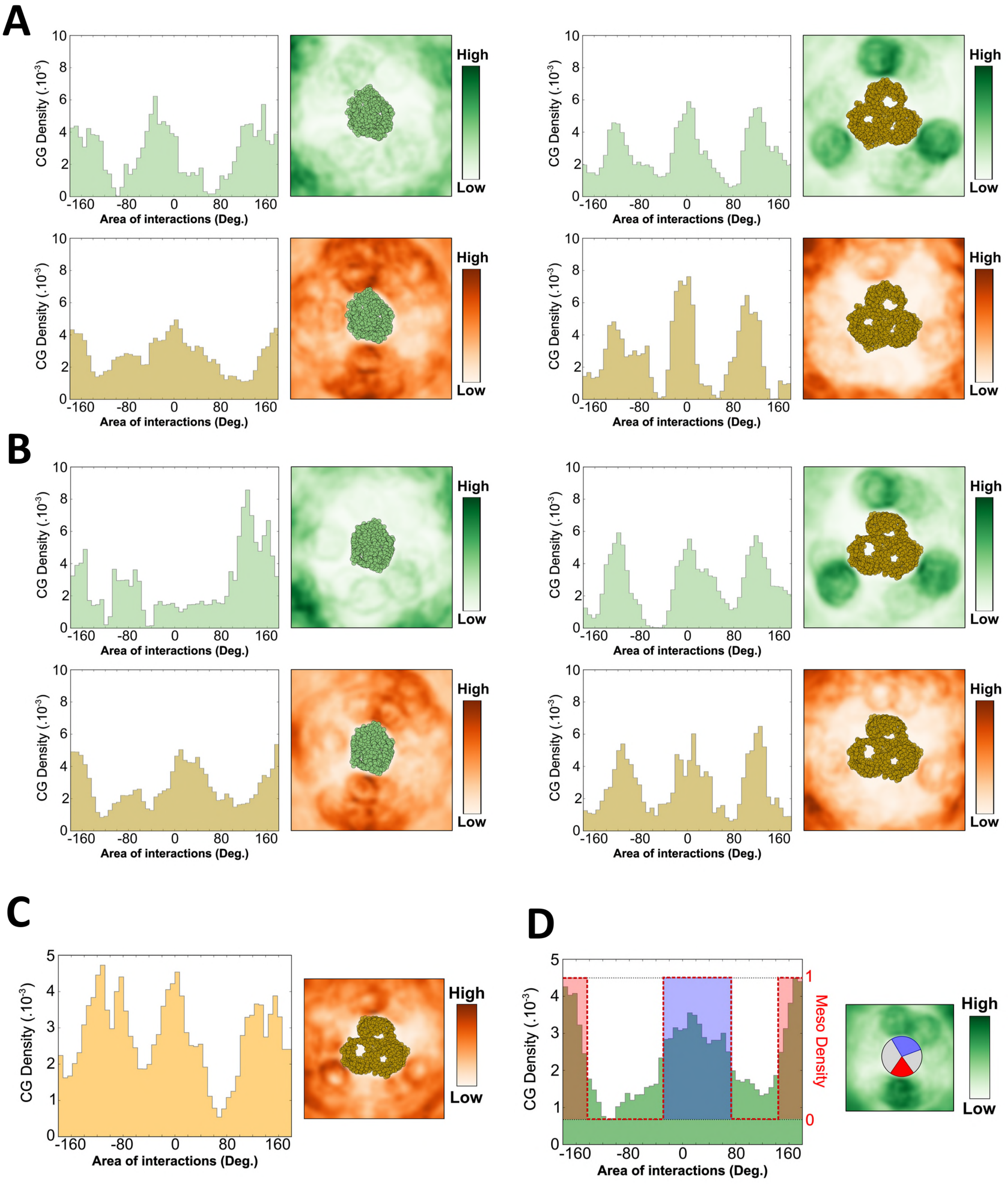

Supplementary Figure 8. Neighbour density around BtuB and OmpF proteins for Mixed systems.

A Density of neighbours to BtuB (left) and OmpF (right) for the Mixed313 systems. The BtuB protein densities are depicted in green and OmpF trimer densites in orange. On the left of each density image is the density shown as a function of the angle around the protein. The densities are higher for BtuB-OmpF interactions due to the system construction favouring this type of interactions. **B** Density of neighbours for the Mixed323 systems. **C** OmpF density for the OmpF323 system. The OmpF-OmpF density is more marked than for mixed systems (A and B) but overlaps with densities seen in these systems. **D** Density of a neighbouring interacting BtuB molecule as a function of angle around a central BtuB molecule, from the BtuB323 simulation. Results for the BtuB313 simulation are shown in Figure 5.

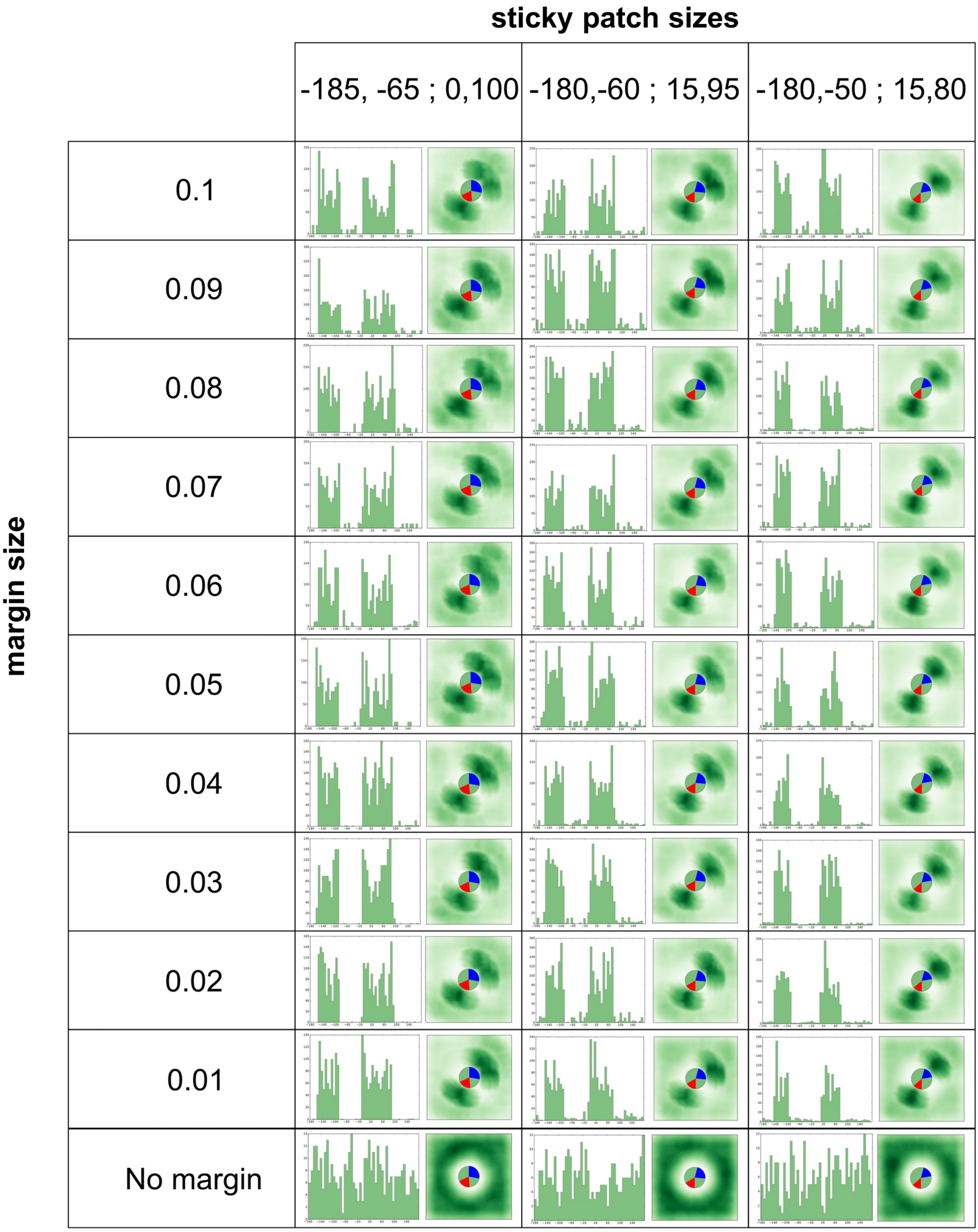

Supplementary Figure 9. BtuB neighbour density evolution as a function of the margin and sticky patch size for 144-BtuB mesoscale simulations.

In order to parameterise the margin size and refined the sticky patches sizes, we launched several mesoscale simulations of 20 µs contained 144 BtuB and assess the density of neighbours around BtuB protein.

sticky patch sizes

margin

	-185, -65 ; 0,100	-180,-60 ; 15,95	-180,-50 ; 15,80
0.10	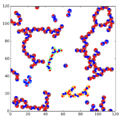 >16	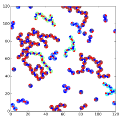 >16	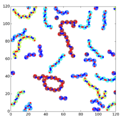 15
0.09	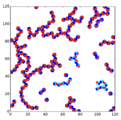 >16	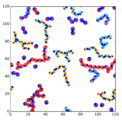 13	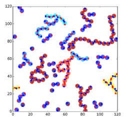 >16
0.08	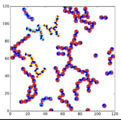 >16	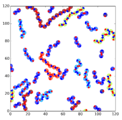 >16	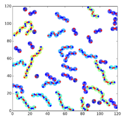 9
0.07	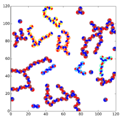 >16	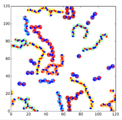 15	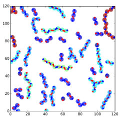 16
0.06	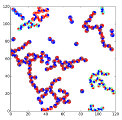 >16	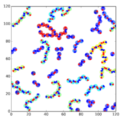 14	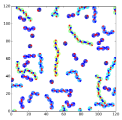 9
0.05	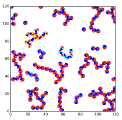 >16	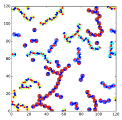 >16	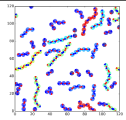 14
0.04	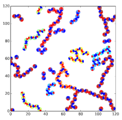 >16	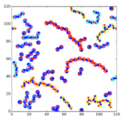 14	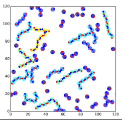 10
0.03	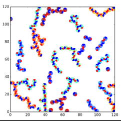 >16	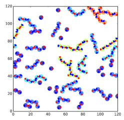 12	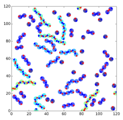 8
0.02	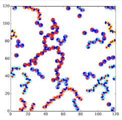 >16	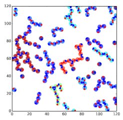 >16	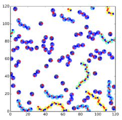 10
0.01	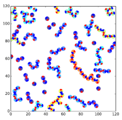 12	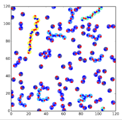 10	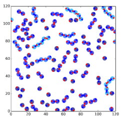 5
No margin	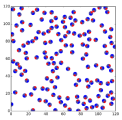 1	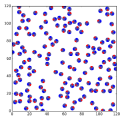 1	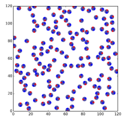 1

Supplementary Figure 10. Cluster evolution as a function of the margin and sticky patches sizes for 144-BtuB mesoscale simulations.

In order to parameterise the margin size and refined the sticky patches sizes, we launched several mesoscale simulations of 20 µs contained 144 Btubs and assess the clustering of BtuB proteins. The numbers each cell of the table give the number of proteins in the largest cluster. The margin size is the proportion of the protein radius that allowed to overlap with an adjacent protein.

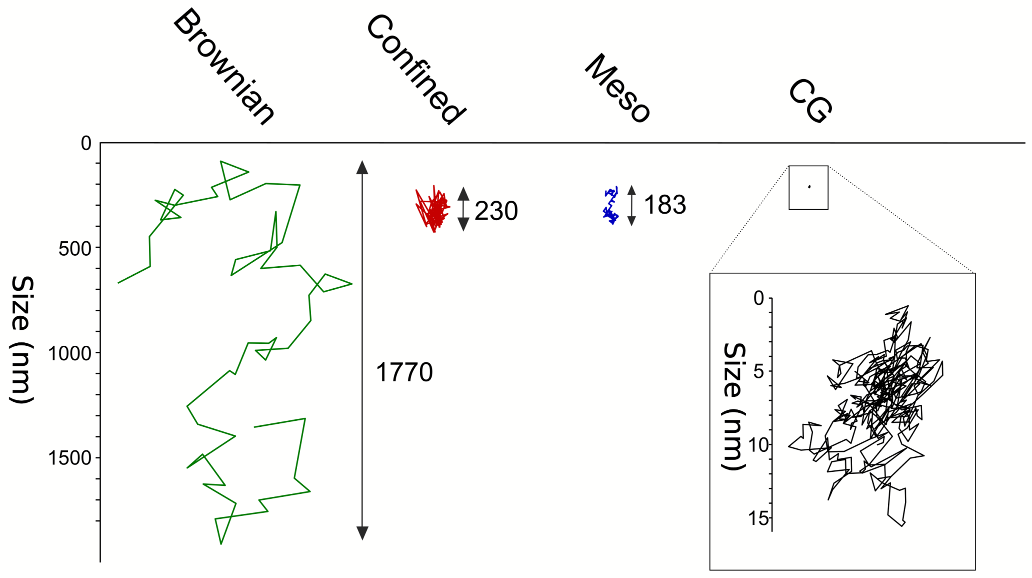

Supplementary Figure 11. Spatial extent of mesoscale simulations in comparison with in vitro and CG-MD trajectories.

This may be compared to the main figure 4A, with the corresponding spatial extent of the mesoscale simulations added. BtuB trajectories are shown from the mesoscale BtuB_interfaces simulation (blue trace), alongside two selected in vitro BtuB trajectories (Brownian, green; confined, red) and a trajectory (black) selected from the BtuB323 CG-MD simulation. Dimensions of the mesoscale and in vitro trajectories are shown in nanometres. Note that the in vitro trajectories are collected over a timescale of seconds, the mesoscale simulation is over a timescale of 24 ms, and the CG-MD trajectory encompasses 20 µs.

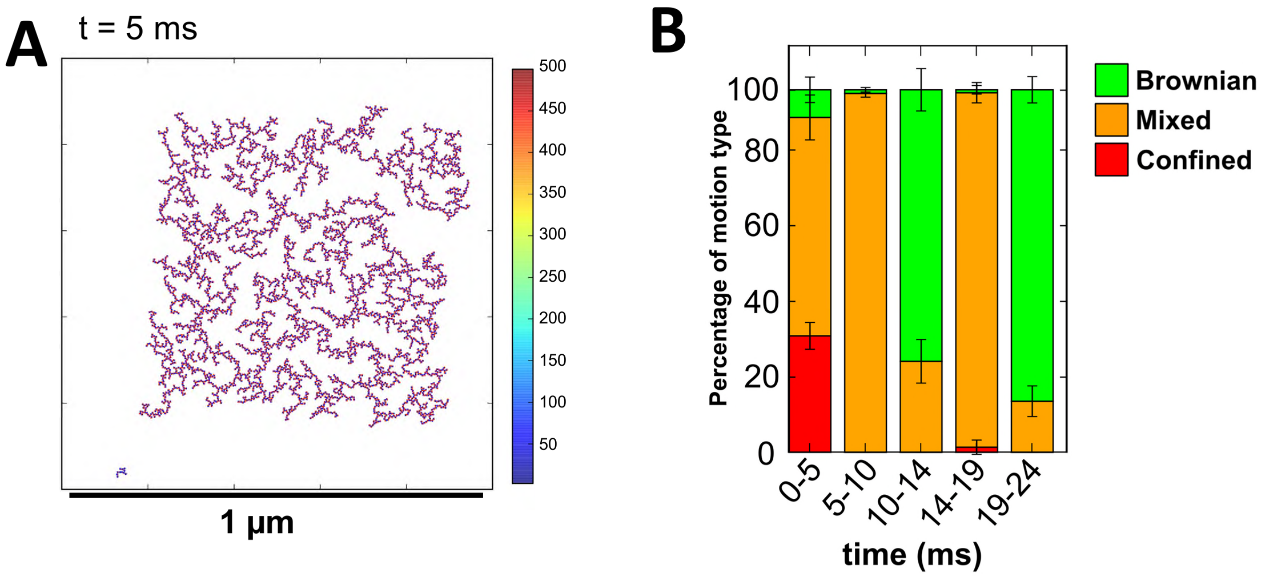

Supplementary Figure 12. Mesoscale simulation with no specific interfaces.

A Snapshot at 5 ms of the No_specific_interfaces mesoscale simulation of a 0.5 μm patch of membrane containing 4900 proteins in a 1 μm^2 box (see Table 1). The simulation box dimensions (1 μm^2) correspond to those of an experimentally observed OMP island. Proteins are coloured according to cluster size, as indicated on the colour scale bar. **B** Fraction of different classes of diffusional motion (Brownian, green; confined, red; or mixed Brownian and confined, orange) for a random sample of 100 from the 4900 proteins in the mesoscale simulation as a function of time. Error bars show the standard deviation given by taking five repeat resamples of 100 proteins.

Supplementary Table 1. Power law fits for BtuB rotations (see Fig. 5a) and translations (see Fig. 5b).

System	Rotation			translation		
	A (°)	*b*	residual	*A* (nm)	*b*	residual
BtuB313	6.11	-0.77	0.006	0.39	-0.20	0.00
BtuB323	6.91	-0.75	0.024	0.45	-0.22	0.01
Mixed313	5.00	-0.67	0.084	0.33	-0.29	0.03
Mixed323	5.99	-0.71	0.165	0.39	-0.32	0.03

Distributions of rotation and translation as a function of the cluster size of the proteins were obtained from the CG-MD simulations and fitted by normal distributions of mean zero and a standard deviation dependent on cluster size. The standard deviation of the rotation (or translation) was plotted with respect to cluster size (Fig 5 a, b) and this was fitted using the equation: $y = Ax^{-b}$, where y is the standard deviation of rotation (or translation) and x, the cluster size.

Supplementary Table 2. Comparison of In Vitro, CG-MD and Meso diffusion coefficients for BtuB

System	D ($\mu m^2\ s^{-1}$)	
	Brownian	Confined
In Vitro[a]	0.02 to 0.4	0.001 to 0.06
CG-MD: BtuB313[b]	3.4	2.4*
Meso: BtuB_interfaces[c]	0.4	0.2
Meso: No_specific_interfaces[d]	0.4	0.5*
Meso: BtuB_1ms[e]	0.3	< 0.05
Meso: Insert_25[f]	1.3	0.1

[a]Experimental estimates from the in vitro data shown in Figure 3a, for a supported bilayer containing a high area density (ca. 30%) of BtuB.
[b]Values taken from the BtuB313 simulation (see Table 1), estimated over the final 4 μs of the simulation. *Classified by PaTrack as 'Mixed' rather than fully 'Confined'.
[c]Values taken from the Meso BtuB_interfaces simulation (see Table 1), averaged over the final 4.8 ms of the simulation.
[d]Values for the Meso No_specific_interfaces simulation (see Table 1), averaged over the final 4.8 ms of the simulation. *Classified by PaTrack as 'Mixed' rather than fully 'Confined'.
[e]Values taken from the Meso BtuB_1ms simulation (see Table 1), averaged over the final 0.2 ms of the simulation.
[f]Values for the 25 newly inserted BtuB molecules in the Meso Insert_25 simulation (see Table 1), averaged over the final 0.2 ms of the simulation.
Also see Supplementary Figure 5.

Supplementary References

1 Casuso, I. *et al.* Characterization of the motion of membrane proteins using high-speed atomic force microscopy. *Nature Nano.* **7**, 525-529 (2012).

2 Rassam, P. *et al.* Supramolecular assemblies underpin turnover of outer membrane proteins in bacteria. *Nature* **523**, 333-336 (2015).

3 Dosset, P. *et al.* Automatic detection of diffusion modes within biological membranes using back-propagation neural network. *BMC Bioinform.* **17** (2016).

4 Goose, J. E. & Sansom, M. S. P. Reduced lateral mobility of lipids and proteins in crowded membranes. *PLoS Comp. Biol.* **9**, e1003033 (2013).
