# Supplementary material for: How nanoscale protein interactions determine the mesoscale dynamic organisation of bacterial outer membrane proteins
Source: Nat Commun. 2018 Jul 20;9:2846. doi: 10.1038/s41467-018-05255-9 (PMC6054660; doi:10.1038/s41467-018-05255-9)
Supplement: Supplementary file 2 — Description of Additional Supplementary Files [file 41467_2018_5255_MOESM2_ESM.docx]

**Description of Additional Supplementary Files**

File Name: Supplementary Movie 1

Description: Evolution of clusters for the Mixed323 simulation.

File Name: Supplementary Movie 2

Description: Mesoscale simulation of 144 BtuB proteins over 20 µs. Proteins are coloured according to their cluster size (colorbar is shown on Figure 6A).

File Name: Supplementary Movie 3

Description: Mesoscale simulation of 24 milliseconds of a 0.5 x 0.5 µm2 patch of 4900 BtuB proteins in a 1 µm2 box (BtuB_interfaces mesoscale simulation in Table 1).

File Name: Supplementary Movie 4

Description: Mesoscale simulation of 24 milliseconds of a 0.5 x 0.5 µm2 patch of 4900 proteins that have no specific interaction interface in a 1 µm2 box (No_specific_interfaces mesoscale simulation in Table 1).

File Name: Supplementary Movie 5

Description: Mesoscale simulation of 1 millisecond of 2500 BtuB proteins.

File Name: Supplementary Movie 6

Description: One millisecond mesoscale simulation of proteins (coloured dots) inserted into an existing clustered patch of proteins, that was taken from the final snapshot of the 1 ms simulation shown in Supplementary Movie 3. Protein trajectories are shown in blue (unclassified motion), green (Brownian motion) and red (confined motion).
